# An adaptive, energy-efficient and secure routing protocol for zone-related mobile Ad-hoc networks using reinforcement learning

**DOI:** 10.1038/s41598-025-32918-7

**Published:** 2025-12-24

**Authors:** Swati B. Singh, Murtaza Abbas Rizvi, Kanak Saxena, Ramji Gupta, Abhishek N. Tripathi, Niraj Kumar Dewangan

**Affiliations:** 1 Department of Computer Science and Engineering, Rajiv Gandhi Proudyogiki Vishwavidyalaya (R.G.P.V), Bhopal, 462033 India; 2 Department of Computer Science Engineering, National Institute of Technical Teachers Training and Research, Bhopal, 462002 India; 3 Department of Computer Science Engineering, Samrat Ashok Technological Institute, Vidisha, 464001 India; 4 Department of Electronics and Communication Engineering, Parul Institute of Engineering and Technology, Faculty of Engineering and Technology, Parul University, Vadodara, 391760 Gujarat India; 5 School of Electronics Engineering, Vellore Institute of Technology, Vellore, 632014 Tamil Nadu India; 6 Department of Mechatronics, Manipal Institute of Technology, Manipal Academy of Higher Education, Manipal, Karnataka India

**Keywords:** Electrical and electronic engineering, Computer science

## Abstract

The rapid surge of Mobile Ad Hoc Networks (MANETs) stimulates the need for adaptive, intelligent, and secure routing mechanisms to ensure seamless communication in dynamic environments. Traditional routing protocols are battling with security threats such as wormhole attacks that disrupt routing and degrade network performance. To address these challenges, this study outlines a state-of-the-art technique, the Reinforcement Learning-Based Secure Routing Protocol (RLSRP), which leverages adaptive k-hop clustering and deep Q-Networks (DQN) to fine-tune routing decisions dynamically while mitigating security risks. RLSRP unwaveringly measures network condition by evaluating latency variations and anomalies to spot suspicious nodes, thereby enhancing route stability. The protocol implements zone-related clustering where nodes within each zone collaborate to optimise routing paths based on real-time conditions, ensuring energy-efficient communication. Current research investigated deep reinforcement learning methodologies to improve security in zone-related MANETs and ensure efficient data routing in large-scale environments. A detailed simulation-based evaluation depicts the potency of the proposed RLSRP model when compared with other reinforcement learning-based routing protocols. Using a large-scale setup–scaling up to 10 million nodes–with Dask and TensorFlow, the results show that RLSRP consistently outperforms FSSAM, Cluster-RL, and Reputation-based Q-learning in terms of Packet Delivery Ratio (exceeding 99%), reduced latency, and improved energy efficiency. These findings reported RLSRP as a secure and scalable solution for practical MANET routing.

## Introduction

**Fig. 1:**
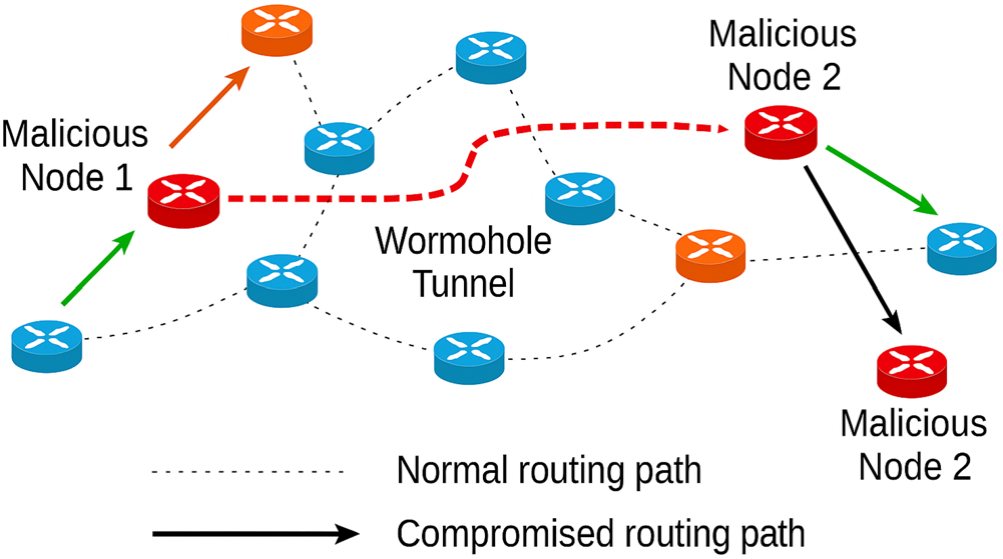
Illustration of a wormhole attack in a Mobile Ad Hoc Network.

Recent years have seen a significant increase in the use of mobile and wireless devices, fundamentally changing how we communicate and interact. From smartphones and wearable gadgets to drones and autonomous vehicles, modern systems heavily rely that can operate independently of fixed infrastructure.These networks must adapt to constantly changing conditions, regulate moving nodes, cope with varying link quality, and conserve limited energy resources. Mobile Ad Hoc Networks (MANETs) provide a practical solution, offering decentralized, self-organizing communication in which each node acts as both a host and a router. Their adaptability renders them applicable across diverse domain, such as disaster response, military operations, vehicular networks, and temporary event networks, where conventional infrastructure is either unavailable or insufficient. Nevertheless, as this technology has evolved, traditional routing protocols, such as ad hoc on-demand distance vector (AODV) and dynamic source routing (DSR), consistently count on,statically defined routes and fail to adapt effectively to frequent topology changes or detect security threats. Vulnerabilities such as wormhole and blackhole attacks pose serious risks, compromising the integrity, availability, and confidentiality of data transmitted through the network. Among these, wormhole attacks constitute one of the most severe threat in MANETs. Malicious nodes create a tunnel (wormhole) between two far-apart nodes in the network, deceiving legitimate nodes into believing that this tunnel offers the shortest path. As a result, routing is disrupted, and henceforth allows attackers to seize control over packet forwarding This enables them to eavesdrop on traffic, delay transmissions, or drop packets, ultimately degrading network performance. As illustrated in Figure 1, two colluding malicious nodes (Node A and Node B) establish a high-speed tunnel, intrigues most of the traffic through themselves and leaving the network vulnerable to manipulation. Detecting such attacks is extremely challenging, particularly in resource-constrained environments. Existing countermeasures typically rely on trust-based models or cryptographic authentication, nevertheless, these techniques introduce significant computational and communication overhead. Reinforcement Learning (RL) techniques, especially Deep Q-Networks (DQNs), have been explored for adaptive routing, as they combine RL with deep neural networks to handle high-dimensional state spaces. This permits adaptive route selection in changing topologies where conventional protocols are ineffective. By constantly learning from network feedback, DQNs improve routing efficiency and adapt to mobility patterns. However, conventional DQN-based methods mainly optimize routing performance and struggle to detect sophisticated attacks such as wormholes, and their scalability degrades in large or highly dynamic networks.

In light of these constraints the rising use of mobile devices and the inherently dynamic characteristics of MANETs showcase the imperative need for a secure, intelligent, and energy-efficient routing protocol. Existing solutions predominantly address one aspect, such as security or energy efficiency, but impose computational and communication overheads that render them unsuitable for deployment in large-scale, resource-constrained networks. Conventional reinforcement learning (RL)-based approaches, including those relying on Deep Q-Networks (DQN), can successfully learn optimal routing paths Nonetheless, these approaches often face challenges when implemented in extensive or dynamically changing networks,their reliance on static assumptions or high computational demands reduces practicality in resource-constrained MANETs. Hence, there is a strong need for an adaptive and lightweight solution that not only balances security, performance, and energy efficiency but also scales effectively for large and highly dynamic MANET deployments.

Security is another critical concern. Existing methods generally count on trust-based models or cryptographic authentication, which, although effective, introduce considerable computational or communication overhead and are impractical for large-scale or highly dynamic networks. Hence, there is a strong need for an adaptive and lightweight solution that not only balances security, performance, and energy efficiency but also scales effectively for large and highly dynamic MANET deployments. To address these challenges, we introduce a state-of-the-art method in our proposed work: the Reinforcement Learning-Based Secure Routing Protocol (RLSRP), which meets these requirements. Detailed mechanisms for wormhole attack detection are presented in Section 3. RLSRP harnesses an adaptive k-hop clustering mechanism for efficient node organisation and DQN-based decision-making to identify optimal routes while mitigating wormhole attacks.. Our approach dynamically scrutinises network conditions by observing latency variations and anomalies to spot spurious nodes.

Reinforcement Learning (RL) is a subset of machine learning that combines advanced methodologies such as Deep Reinforcement Learning (DRL) with algorithms such as Deep Q-Networks (DQN) and furnishes feasible solution for optimizing routing decisions derived from environmental feedback. This facilitates adaptive route selection, adeptly addressing challenges triggered by network instability while supporting intelligent decision-making. By incorporating zone-based organization, deep reinforcement learning, and scalable simulation tools (Dask, TensorFlow, and NetworkX), RLSRP signifies a considerable improvement in a secure, resilient, and scalable path for MANETs. The protocol competently maintains a balance between security, energy consumption, and routing efficiency, even in networks with a capacity of tens of millions of nodes. Its adaptive nature enables it to quickly adapt to frequent changes in network topologies and emerging threats, ensuring reliable communication in highly dynamic and dense environments. The main contribution of the proposed work, viz. 1. Our proposed RLSRP, a Reinforcement Learning-Based Secure Routing Protocol that uses Deep Q-Networks (DQN) to enable real-time, adaptive, and secure routing within zone-related Mobile Ad Hoc Networks (MANETs). This provides the capability to quickly learn and adapt to dynamic network conditions, augmenting static or heuristic-based protocols.

2. The protocol adopts a latency-based wormhole detection mechanism that identifies abnormal delays in packet dissemination to uncover wormhole attacks. This approach cleverly evades the computational and communication overhead of conventional cryptographic or trust-based methods, allocating efficient and lightweight security solutions appropriate for resource-constrained MANET environments. An adaptive k-hop clustering mechanism is introduced to reduce routing overhead by effectively segmenting the network into zones. This clustering aids scalable intra- and inter-zone communication, weakening control message flooding, thereby refining network efficiency.

4. Reinforcement learning-driven decision-making is paired with Q-values modifies, this adaptive learning framework augments route reliability and security concurrently, addressing gaps among numerous existing zone-based routing protocols.

5. Analytical and simulation-based evaluations depicts that RLSRP delivers a higher packet delivery ratio (PDR),lower latency, and reinforces energy efficiency relative to conventional protocols. This endorses RLSRP as a robust and versatile solution balancing security, performance, and energy consumption for large-scale, dynamic MANET deployments.

The remainder of this paper is structured as follows. Section II reviews related work on RL-based secure routing protocols in MANETs.In Section III, we present the system model, including network assumptions, threat model, and design overview. Section IV details the proposed Reinforcement Learning based secure routing protocols, and thoroughly describes the working of the proposed algorithm strategy. Section V describes the simulation setup, performance metrics, and comparative evaluation with existing RL-based routing protocols. Finally, Section VI concludes the paper and outlines potential future research directions.

## Literature review

Mobile Ad-hoc Networks (MANETs) face significant security challenges, particularly wormhole attacks that can disrupt network communication. Various techniques have been proposed to mitigate these threats, including trust-based mechanisms, anomaly detection, and machine learning-based approaches, we present an in-depth analysis of existing reinforcement-based learning protocols and traditional routing protocols such as the ad hoc on-demand distance vector (AODV), dynamic source routing (DSR)^1^ have been widely used in MANETs. However, these protocols are vulnerable to security threats as they rely on simple hop-based routing mechanisms. Optimized Link State Routing (OLSR)^2^ improves routing efficiency but lacks robust security measures. Furthermore, we examine current countermeasures against wormhole attacks, focusing on their effectiveness while rigorously addressing their limitations and the remaining gaps. In addition to traditional routing protocols, recent studies have proposed mechanisms intended to strengthen route discovery and manage link failures. For instance^3^ discusses the Zone-Based Route Discovery Mechanism (ZRDM) blend with a Link Failure Prediction Mechanism (LFPM) to improve source routing in MANETs. Their approach focuses on dividing the network into zones for efficient route discovery and analysing potential link failures to improve route stability. While this mechanism enhances routing performance and reliability, it does not integrate any security features such as wormhole attack detection, nor does it employ reinforcement learning for adaptive decision-making. This establishes a pathway for protocols like the proposed RLSRP, which incorporates wormhole detection and leverages deep Q-learning to provide secure and energy-efficient routing

Imran et al.^4^ proposed a wormhole detection mechanism that provides comprehensive analysis of wormhole attacks in Mobile Ad Hoc Networks (MANETs), highlighting their significant threat to routing processes without compromising nodes. It investigates several effective detection and prevention strategies, including the Wormhole Attack Prevention Algorithm (WAP), Hop-Count Analysis, and Trust-Based Models. Additionally, it also discussed serious challenges that wormholes pose, such as creating deceptive routes and enabling Denial-of-Service attacks. It also emphasises that many of them involve extra hardware; certain algorithms can notably reduce costs and overhead, paving the way for more resilient and cost-effective strategies to protect MANETs.The paper offers a comprehensive analysis of wormhole attacks in Mobile Ad Hoc Networks (MANETs), highlighting their significant threat to routing processes without the necessity of compromising any nodes. It delves into several robust detection and prevention strategies, including the Wormhole Attack Prevention Algorithm (WAP), Hop-Count Analysis, and Trust-Based Models, which are designed to effectively minimize the presence of these attacks.

Ozen et al.^5^ addresses the challenges of route stability and limited energy in Mobile Ad Hoc Networks (MANETs), which are commonly driven by frequent topology changes and node failures. To overcome these issues, the authors propose an energy-efficient routing protocol that integrates Ant Colony Optimization (ACO) with the traditional AODV protocol.In the proposed method, pheromone values guide route selection depending on multiple node metrics, including residual energy, energy drain rate, hop count, and node speed, ensuring the discovery of more reliable and stable routes. This hybrid approach lessens redundant traffic, minimizes energy consumption, and enhances the overall lifetime of the network.Simulation results describes that the proposed algorithm excel standard AODV in key performance metrics such as packet delivery ratio, throughput, end-to-end delay, packet loss ratio, and network lifetime, showcasing its effectiveness in improving both stability and energy efficiency in MANETs..

hassan et al.^6^ proposed LSTMT-LAR, a routing protocol that employs LSTM-based trust prediction with Location-Aided Routing (LAR) to strengthen security in MANETs. Using a 13-feature behavioral model, the protocol evaluates node trustworthiness in real-time to detect and isolate malicious nodes. In simulations with varying node densities, LSTMT-LAR achieved a high Packet Delivery Ratio while prolonging competitive end-to-end delay and reduced energy consumption. Regardless of its effectiveness, the authors note that challenges remain in scalability, resilience under colluding attacks, and the trade-off among trust computation and energy consumption, which open up opportunities for further research.

Pramodh Krishna D. et al. (2024)^7^ proposed a hybrid deep learning model for flooding attack detection in MANETs, which struggles with high computational complexity as a consequence of integrating CNN, LSTM, and GRU architectures. It depend truly on MATLAB simulations, which inadequately portray the dynamic and unpredictable nature of real-world MANET environments.The model is devoid off adaptability to evolving attack strategies and entails ongoing retraining and fine-tuning efforts to sustain its detection performance. Consequently, its practical deployment remains an open research area. In contrast, our proposed RL-based secure and energy-efficient routing model utilizes a lightweight Q-Routing mechanism with adaptive K-hop clustering to enable real-time learning, scalability, and robustness. This enables our model more adaptive, energy-efficient, and suitable for large-scale MANET applications.

Bhatti et al. (2024)^8^ indicated a heuristic-based method for uncovering and quarantine wormhole nodes in wireless ad hoc networks.by observing post-wormhole actions such as TTL tampering, packet replaying, and looping. Their method achieved a high detection accuracy of 98–99% without relying on additional hardware, proving computationally efficient and lightweight. Nevertheless the approach is reactive in nature, detecting wormholes only after an attack has occurred, and depends on static thresholds that limit adaptability to mobile or intelligent attackers. Its scalability and effectiveness also decline in highly dynamic MANET environments. Although the technique performs well for post-attack detection, it lacks proactive learning and predictive defense capabilities. Consequently, this work is still an open area of research, encouraging further exploration into adaptive, intelligent, and self-learning mechanisms for proactive wormhole attack prevention and secure routing in MANETs.

Rathod, J.A^9^ The author proposed a hybrid routing mechanism that combines AODV (Ad hoc On-Demand Distance Vector) and MBOMRP (Multi-Path Byzantine OLSR) to establish multiple secure paths between source and destination nodes. A hybrid cryptographic scheme was further employed to securely transmit fragmented data, thereby improving packet delivery, bandwidth utilization, and overall network security. However, the approach still suffers from limitations such as routing overhead due to frequent cluster head re-election, increased control complexity, and higher energy consumption from cryptographic operations. These limitations indicate that the study remains an open research area for future researchers to address issues of scalability, lightweight security, and efficiency in highly dynamic MANET environments.

Ali, Z.H et al.^10^ proposed a hybrid routing mechanism that combines AODV (Ad hoc On-Demand Distance Vector) and MBOMRP (Multi-Path Byzantine OLSR) to establish multiple secure paths between source and destination nodes. A hybrid cryptographic scheme was subsequently adopted to securely transmit fragmented data, thereby improving packet delivery, bandwidth utilization, and overall network security. Nevertheless, the approach still suffers from limitations such as routing overhead due to frequent cluster head re-election, increased control complexity, and higher energy consumption from cryptographic operations. These limitations indicate that the study remains an open research area for future researchers to address issues of scalability, lightweight security, and efficiency in highly dynamic MANET environments.

Abdullah, Ako et al.^11^ proposed the E-AODV protocol, which demonstrates a clear improvement over traditional AODV and SBADR by using a multi-metric approach for route selection, enhancing stability, throughput, and reducing delay and routing overhead. They introduced the Route Stability Factor as a key strategy for selecting reliable routes in dynamic MANETs. While the protocol relies on accurate link lifetime estimation, it provides a strong and promising foundation for multi-metric routing approaches, with potential for even better performance under diverse network conditions.

Jinqiao Wu et al.^12^ proposed Q-learning-based Traffic-Aware Routing (QTAR) has been introduced as an RSU-assisted protocol that combines geographic forwarding with road segment traffic awareness. QTAR employs V2V Q-learning within road segments and R2R Q-learning at intersections to improve the delivery ratio and minimize end-to-end delay under diverse densities. Although the protocol represents a notable amendment to RTAR and GyTAR, it is heavily based on the deployment of RSUs at intersections, which makes it infrastructure dependent and inappropriate. Furthermore, QTAR primarily optimizes routing performance in terms of delivery ratio and delay, but does not address other critical aspects such as security, energy efficiency, or large-scale scalability. In contrast, our proposed approach obliterates the dependence on RSUs by leveraging adaptive K-hop clustering and zone formation, enabling fully distributed operation. Furthermore, by integrating reinforcement learning with trust-based anomaly detection and energy-sensitive route selection, our model ensures secure and efficient communication, even in large-scale networks with millions of nodes. This makes our protocol a more comprehensive solution to the challenges of VANET routing compared to QTAR.

Zohaib Hassan et al.^10^ proposed, integrating Software-Defined Networking (SDN) with fog computing to strengthen the capabilities of Vehicular Ad Hoc Networks (VANETs). The system incorporates an energy-efficient and QoS-aware geographical routing protocol designed to supervise data transmission. The simulation results show that the approach achieves notable refinement. These findings underscore the virtue of the protocol in optimizing data transmission and supporting real-time communication requirements. Nevertheless, the reliance on simulation for evaluation highlights a hindrance shared with most studies in this domain; practical validation under large-scale deployment and highly dynamic traffic conditions remains a open research for future exploration.

Nai-Wei Lo et al.^13^ investigated the impact of scalability in large-scale Mobile Ad Hoc Networks (MANETs), focusing on how increased network size influences performance and security. While their study addressed the challenges of efficient routing in dynamic environments, they also highlighted energy consumption as a significant concern, emphasizing the need for optimized routing mechanisms. They introduced the CBDAODV (Cooperative Blackhole Detection AODV) mechanism to defend against cooperative blackhole attacks. According to the author, the survey is meant to be a helpful reference for researchers and practitioners interested in implementing RL-based routing in network environments.

Qiu Xiu-feng et al.^14^ In this paper, an MTSR (Multipath Trust-Based Secure Routing) was proposed, a routing protocol that addresses security issues in ad hoc networks. The authors highlight the vulnerability of ad hoc networks to various attacks, with a particular focus on the wormhole attack. MTSR is based on AODV and SAODV protocols, combining cryptography and trust mechanisms. The authors conclude that MTSR offers a practical and effective solution for secure routing in ad hoc networks. However, findings reported that some parameters, such as computational overhead and the size of the network, are still hurdles as far as security is concerned; hence, it holds major prospects as a future research direction.

Muhannad Tahboush et al.^15^ (HWAD) proposed a significantly enhanced detection efficiency. HWAD effectively addresses key challenges in existing solutions, such as high delays, low throughput, and energy consumption, a provides round trip time (RTT) and packet delivery ratio (PDR) for in-band detection. Implemented within the AODV routing protocol, its performance has been examined through comprehensive NS-2 simulations. The HWAD algorithm performs well on detecting both in-band and out-of-band wormhole attacks without requiring specialized hardware or middleware, thus minimizing energy usage. The results clearly show that HWAD outperforms existing methods. Future research should focus on improving detection techniques and addressing energy consumption issues in large network environments. The findings reported in our study reveal a significant gap in current detection methodologies, which often rely on specialized hardware and incur high communication overhead. HWAD offers a more efficient solution. It aims to improve detection accuracy across various network conditions, with future studies set to enhance its application in expansive topological areas while addressing the energy limitations of mobile nodes.

In this section, we present an in-depth study of existing work on secure routing in Mobile Ad Hoc Networks (MANETs) and the application of reinforcement learning in network security.

“Security is a major concern in MANETs”, due to their decentralized nature. Traditional security models have struggled to prevent blackhole, wormhole, and Sybil attacks.

Maros Baumgartner et al.^16^ The integrated algorithm leverages decentralized blockchain technology and deep neural networks (DNNs) to detect the legitimate nodes for routing based on historical behavior and real-time network conditions. Security vulnerabilities remain a concern with the integration of blockchain. Although blockchain improved transparency and trust, it also introduces potential security vulnerabilities, which continue to be a critical area of concern. However, the author acknowledges that further refinement is being sought, specifically in examining the number of hidden layers and neurons in the DNN, which is likely to be a future research direction.” This encourages both scholars and practitioners to explore innovative solutions that advance the development of secure and efficient routing protocols.

Several investigations have been carried out to present a comprehensive review of attack models and mitigation strategies in wireless networks. Also, trust-based mechanisms focus their potential on improving network security. Findings suggest that AI-based cybersecurity methods pose potential promise and should be further explored to effectively deal with the evolving threats in wireless network environments. Efficient Security Mechanisms for Routing Protocols

Yih-Chun Hu et al.^17^, the authors present security mechanisms for routing protocols. These mechanisms are based on cryptographic techniques and can be used as cornerstones for securing routing protocols and focusing on efficiency and security against specific attacks. Nodes claiming longer distances than actual can lead to suboptimal routing, as traffic may be directed through longer paths unnecessarily; however, this method provides a valuable insight to refine routing protocols. Results illustrate that the proposed method performed well.

Reinforcement learning (RL) has been increasingly applied to dynamically optimize routing decisions^18^. RL introduced foundational concepts that later inspired the use of Deep Q-Networks (DQN) for routing optimization. Moreover, Several studies have leveraged RL for MANET security.

Mayadunna et al.^19^ proposed a reinforcement learning model, specifically Q-learning. This approach immensely detects malignant nodes in mobile ad hoc networks and implements an RL model using Network Simulator 3 (NS-3). This evaluation provides evidence of the model’s effectiveness in identifying and mitigating the risks posed by malicious nodes. The implementation was carried out, and tests were performed to identify defects, thereby showing dedication to improving the overall reliability of the network. However, there is limited exploration of the model’s performance in large complex networks. This creates a gap in perceiving how the proposed model would be carried out in larger, more complex MANETs, where the dynamics of node interactions and malicious behaviors could differ substantially. However, the study concedes the obstacle posed by the consistent fluctuations in network topology due to node mobility. This opens promising privileges for future researchers to refine the model’s adaptability to dynamic environments and broaden its scope to diverse network environments.

Ohida et al.^20^ discuss that MCRP is a centralized protocol where the Base Station (BS) oversees routing and energy consumption deploys a time tracking mechanism to deter the involvement of malicious nodes. The protocol consists of hop counts, frame types, timestamps, and data fields. It defines algorithms to establish routing paths and detect wormholes. BS uses time ratio thresholds to spot potential wormhole connections.MCRP offers an innovative method for identifying wormhole attacks in wireless sensor networks. However author discussed scalability challenges and susceptibility to other types of attacks. This paper lays the groundwork for future investigations into WSN security and energy management, emphasizing the need for protocols that can evolve with emerging threats while ensuring efficiency.

Deep reinforcement learning (DRL)^21^ has emerged as a powerful tool for secure and adaptive routing. The detection of wormhole and blackhole attacks using DRL has also been explored.

Kiril et al.^22^ Key findings from this study reveal the adoption of deep learning for MANET routing “Deep learning for MANET routing” presents several significant findings regarding the application of deep learning techniques in optimizing routing within Mobile Ad-hoc Networks (MANETs). The author proposed the SPCDNet model demonstrated exceptional performance when tested across a variety of active link configurations. This indicates that the model is robust and can adapt to different network conditions. The results also showed that SPCDNet performance compared to traditional routing schemes, highlighting the usefulness of using deep learning for routing decisions in MANETs. The study also explored a variation of the scheduling and power control problem focused on maximizing throughput under routing constraints. These finding emphasizes the potential for deep learning models to address complex routing challenges in MANETs, paving the way for future research.

Beyond reinforcement learning^23^, AI-driven^24^ security mechanisms have been investigated to improve trust management and intrusion detection.

Fatma Aktas et al.^25^ proposed AI-Enabled Routing, focusing on various security concerns and potential attacks that could compromise the integrity and performance of these systems. The findings suggest that, while AI systems can have the capacity to enhance network performance, they may also introduce vulnerabilities. Reporting these foreseeable risks persists as a barrier as far as the security of routing protocols is concerned.

Despite the advancements in AI-driven security, existing solutions lack a comprehensive integration of deep reinforcement learning for secure zone-based MANET routing. Existing works focus on general security mechanisms but do not specifically address wormhole detection in clustered MANET architectures.

Our proposed Reinforcement Learning Based Secure Routing Protocol (RLSRP) leverages Deep Q-Networks (DQN) to enhance security, optimize route selection, and mitigate attacks dynamically. The aim of the current study is to deal head-on with these problems and propose a more effective and practical approach for detecting and defending against wormhole attacks. Lately, there has been exploration into the use of Deep Reinforcement Learning (DRL) for secure routing. Deep Q-Networks (DQN) have demonstrated notable potential in optimizing route selection by dynamically responding to changes in network conditions. Unlike traditional methods, DQN-based routing consistently learns from network feedback, making it more resilient to attacks.

### Existing solutions and gaps

although existing methods offer skewed solutions to worm hole attack mitigation, they perpetually struggle to adapt to evolving security risks. Several trust-based and anomaly detection methods lean on predefined thresholds, curtailing their effectiveness in a real-world environment. Moreover, traditional protocols cannot efficiently detect wormhole attacks without additional security mechanisms and show limited focus on large network environments. Considering the boundaries of existing approaches, our proposed approach leverages DQN to learn secure and energy efficient routes while dynamically mitigating wormhole attacks. Unlike trust-based models, our approach does not rely on static reputation scores but preferably evaluates network behavior in real time.

## Design of the proposed secure and energy-efficient routing protocol

So far, we have covered the literature review, findings, and research gap, suggesting that traditional routing is struggling and it is cumbersome to handle a large network environment due to frequent topology changes, bandwidth constraints, security vulnerabilities, and so on.“Considering all these significant gaps, thereby this work presents an intelligent routing strategy to begin with the network is divided into adaptive K-Hop clusters, and routing decisions are optimized using reinforcement learning techniques. Likewise, a wormhole detection mechanism is employed to identify and mitigate malicious nodes, ensuring secure routing.

### Proposed wormhole detection mechanism

Once we have comprehended the detrimental impact of wormhole attacks on routing, we now turn to how the proposed mechanism effectively counters them. The DQN-based routing algorithm detects wormhole occurrence by monitoring principal criteria such as::

- Increased transmission delay,
- Elevated packet loss rate,
- Irregular route selection patterns.

**Fig. 2:**
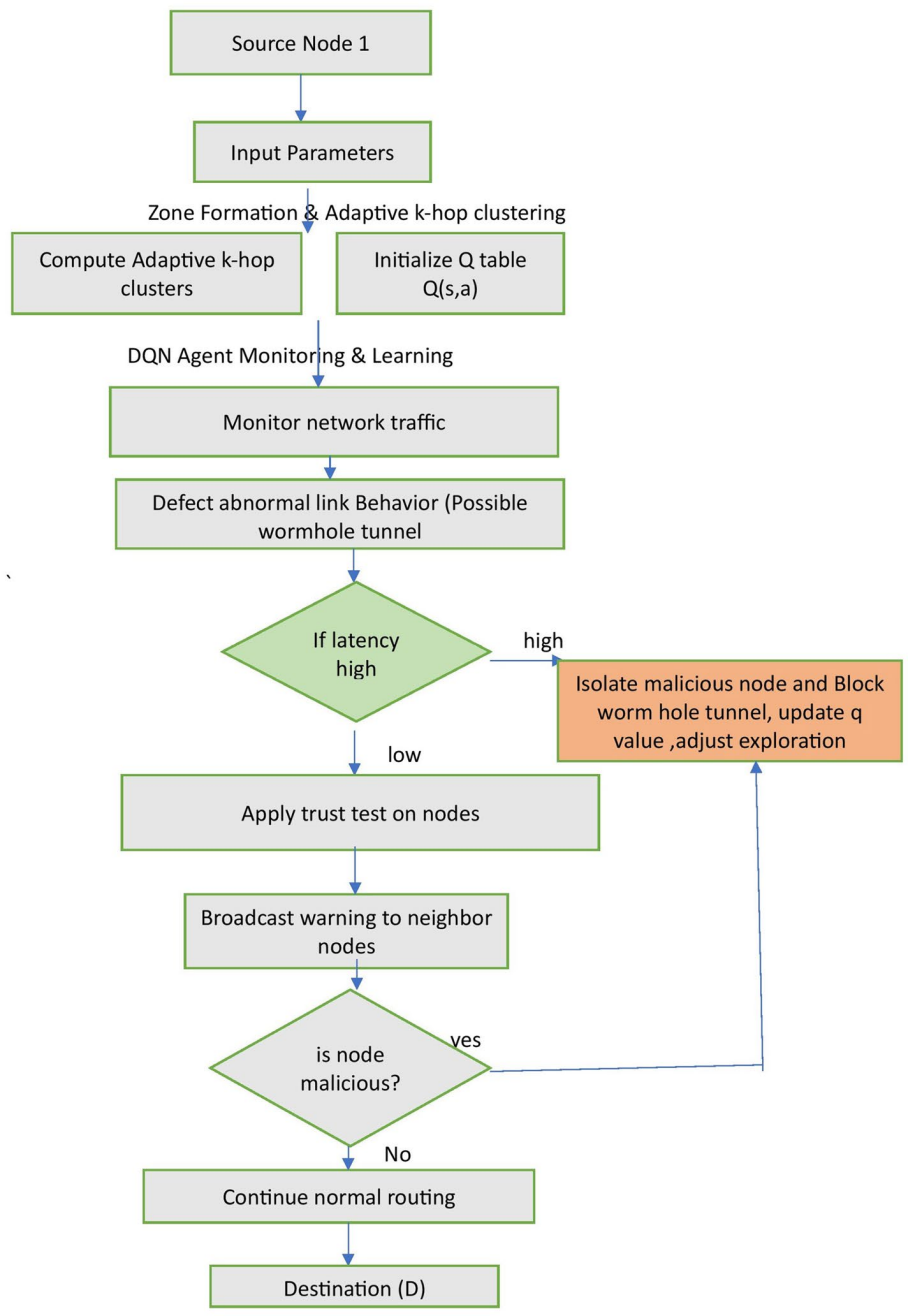
Proposed method for mitigating wormhole attacks and achieving energy-efficient routing.

Once detected, RLSRP segregates untrusted nodes and securely reroutes packets, guaranteeing energy-efficient and resilient communication within Zone-Related MANETs. Figure 2 illustrates the fundamental process of RLSRP in mitigating wormhole attacks by identifying and excluding malicious routes.

The network consists of interconnected nodes that enable data transmission from a source node (S) to a destination node (D)”. The findings are reported to distinguish the most secure and efficient route for data transmission. Trusted nodes adhere to standard routing protocols, preventing the exposure of harmful behaviours. These unwavering nodes play a crucial role in preserving secure and seamless communication. With precision and intelligence, the DQN algorithm detects and chooses the most secure route, linking these trusted nodes to ensure uninterrupted and reliable data delivery. Malicious nodes design wormhole links, deceiving the network by promoting shorter, deceptive paths. This diversion allows data packets to bypass legitimate routes, jeopardizing the integrity of the networks. The DQN meticulously examines delays and packet loss, quickly identifying peculiar routing patterns. by considering the past behaviors of nodes, the algorithm tweaks the most trusted and legitimate route, thereby dynamically quarantining suspicious nodes and paving the way for reliable dissemination of data via substitute routes. Deep Reinforcement Learning coalesces the vast capacity of deep learning in handling complex data with the reinforcement learning paradigm, which learns optimal policies through interaction with the environment. In the context of routing in Zone-Related MANETs, DQNs foster the learning of optimal paths based on various performance metrics, such as node trust scores, network delay, and the reliability of neighbouring nodes. However, traditional routing protocols probably lack the capacity for adaptive learning; instead, they utilize overly simplistic models that cannot dynamically adapt to the evolving state of the network. This research reported a DRL framework that employs an adaptive approach, enabling the network to learn from prior experiences and dynamically choose routes to maximize the likelihood of successful dissemination. The dynamic learning capabilities embedded in DQNs provide a substantial gain in optimizing routing decisions, particularly in environments where nodes repeatedly enter or leave the network. Unlike prior works Instead of focusing solely on energy efficiency or security independently, this work introduces RLSRP – a zone-Related routing protocol that leverages Deep Q-Learning design to address both aspects hence achieving energy-aware route selection and security against wormhole attacks simultaneously. The novelty lies in integrating reinforcement learning into a zone-based MANET architecture while also incorporating proactive wormhole detection. This unified framework has not been previously explored in zone-related MANET literature.The novelty lies in integrating reinforcement learning into a zone-based MANET architecture while also incorporating proactive wormhole detection. This unified framework has not been previously explored in zone-related MANET literature.

To implement this unified framework, the network must first be initialized and represented as an underlying graph structure, which then serves as the foundation for clustering, routing, and security operations.

### Network initialization

The MANET is represented as an undirected graph:

1 $$\begin{aligned} G = (V, E) \end{aligned}$$

Equation 1 Graph representation of the mobile ad hoc network, where $G = (V, E)$ denotes the network, with $V$ as the set of mobile nodes and $E$ as the wireless communication links between them.

**Fig. 3:**
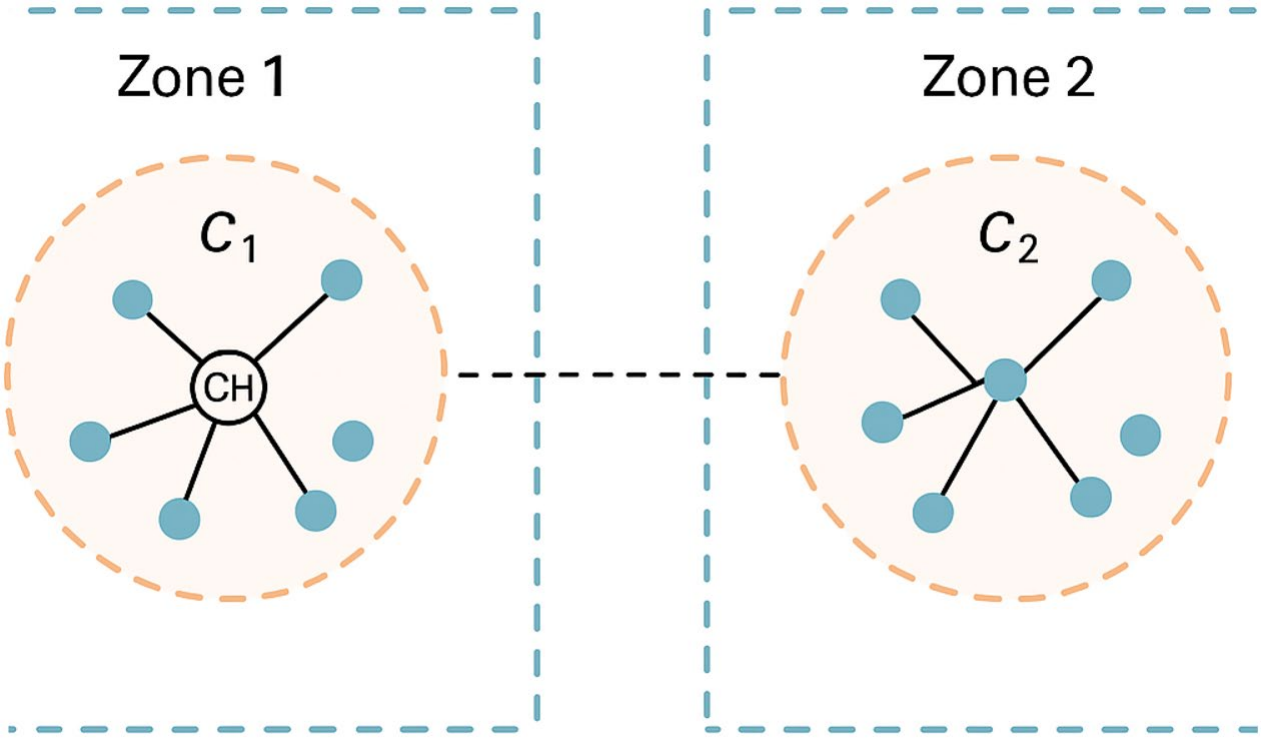
Zone formation in a MANET, where each zone (Zone 1 and Zone 2) contains a cluster $C_1$ and $C_2$, respectively. Each cluster is managed by a Cluster Head (CH), facilitating intra-zone communication and enabling hierarchical routing.

In this phase, the network is initialized. Each node $v_i \in V$ determines its location using a Global Positioning System (GPS). The network is partitioned into non-overlapping zones $Z_j$. These zones are represented by the set $Z = \{Z_1, Z_2, \dots , Z_m\}$, where the union of all zones equals the entire set of nodes in the network, see Figure 3:

2 $$\begin{aligned} Z = \{Z_1, Z_2, \dots , Z_m\}, \quad \bigcup _{j=1}^{m} Z_j = V \end{aligned}$$

Equation 2 Partition of the set of mobile nodes $V$ into non-overlapping zones $Z = \{Z_1, Z_2, \dots , Z_m\}$, where the union of all zones equals the entire set of nodes, $\bigcup _{j=1}^{m} Z_j = V$. As shown in Equation 3. Each zone has a designated Zone Head (ZH) screen based on:

3 $$\begin{aligned} ZH_j = \arg \max _{v_i \in Z_j} f(v_i) \end{aligned}$$

where $f(v_i)$ is a function considering node degree, residual energy, and stability. As depicted in Equation 4. Within each zone $Z_j$, nodes form clusters managed by Cluster Heads (CHs). The clustering follows an adaptive K-hop strategy to form a structured topology. where $C_k^j$ represents the set of nodes $v_i$ within the hop threshold $k(v_i)$ from their corresponding Cluster Head $CH_j$, and $d(v_i, CH_j)$ is the shortest path distance between node $v_i$ and $CH_j$.

4 $$\begin{aligned} C_k^j = \{ v_i \mid d(v_i, CH_j) \le k(v_i) \}, \quad v_i \in Z_j \end{aligned}$$

To enable scalable and stable communication in zone-based MANETs, each node calculates an adaptive hop threshold as shown in Equation 5:

5 $$\begin{aligned} k(v_i) = \alpha \cdot D + \beta \cdot M \end{aligned}$$

where *D* represents node density, *M* denotes node mobility, and $\alpha$, $\beta$ are weight factors. A higher density increases cluster size, while high mobility reduces it to maintain stability. The routing path is structured hierarchically, as illustrated in Equation 6:

6 $$\begin{aligned} v_i \rightarrow CH_i \rightarrow ZH_i \rightarrow ZH_j \rightarrow CH_j \rightarrow v_j \end{aligned}$$

ensuring secure and efficient inter-zone communication.

Data transmission between nodes pursues a hierarchical routing link through CHs and ZHs.This adaptive clustering approach improves scalability, load balancing, and routing efficiency, making it feasible to manage over 10 million nodes. To deal with such large-scale networks, the system employs sparse adjacency lists for memory efficiency, hierarchical clustering for localized communication, and parallel processing (TensorFlow/Dask, NetworkX, GPU acceleration) for real-time routing decisions. Additionally, event-driven updates ensure restricted to moving nodes recompute their links, minimizing computational overhead, while Deep Q-Networks (DQN) optimize routing paths to reduce delay and overhead.

**Fig. 4:**
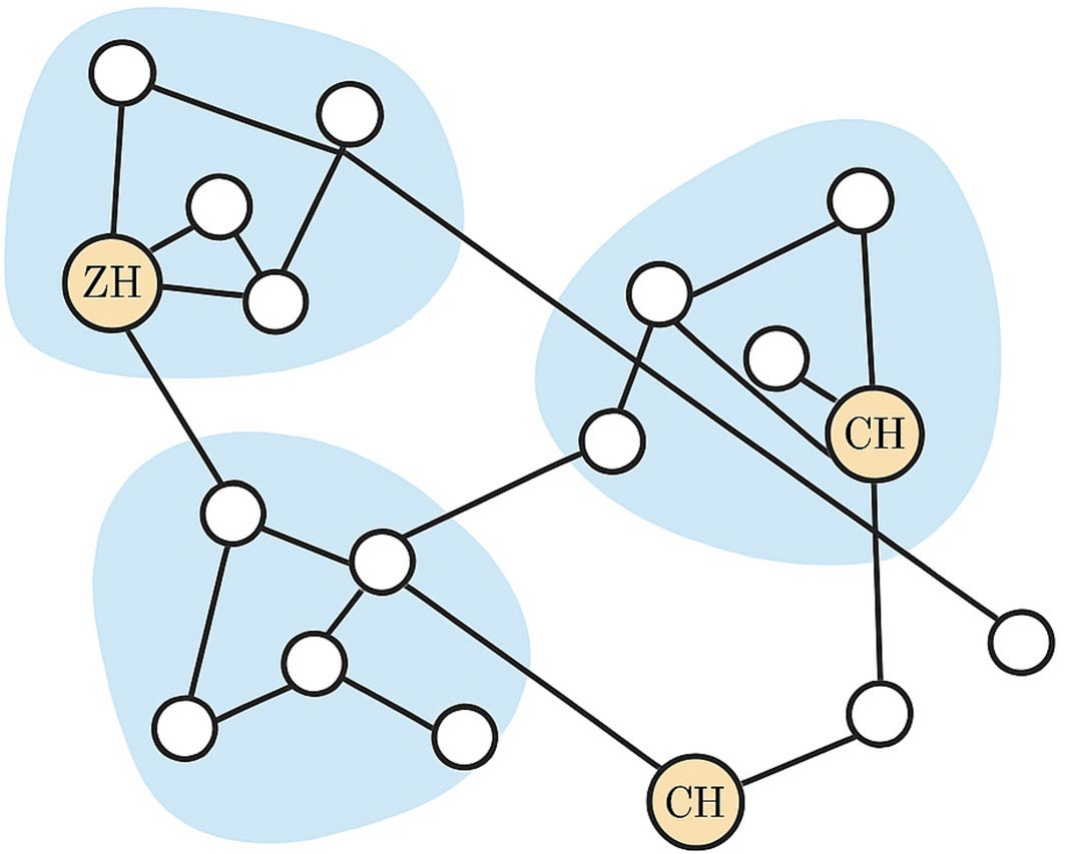
Network model of the proposed MANET system. The network is divided into multiple zones, each managed by a Zone Head (ZH). Within zones, adaptive K-hop clustering forms clusters with Cluster Heads (CH). The hierarchical routing path is illustrated, where data travels from source node $\rightarrow$ CH $\rightarrow$ ZH $\rightarrow$ ZH of the destination zone $\rightarrow$ CH $\rightarrow$ destination node.

The proposed network model for the Zone-Based Mobile Ad Hoc Network (MANET) segments the entire network into multiple zones. Each zone is coordinated by a Zone Head (ZH), which oversees for maintaining intra-zone communication and overall zone-level management. Within each zone, smaller clusters are formed, each led by a Cluster Head (CH). The CHs ensure efficient routing among cluster members and report to the ZH for inter-zone communication. The hierarchical structure improves scalability and reduces routing overhead by dividing routing responsibilities. Intra-cluster communication is handled locally by CHs, while inter-zone routing decisions are made via ZHs. This design diminishes energy consumption, enhances load distribution, and strengthens security by localizing control and enabling anomaly detection at both cluster and zone levels. As illustrates in Figure 4, Nodes (white circles) represent mobile devices contributing in the MANET. CHs (orange circles marked CH) manage their respective clusters. ZH (orange circle marked ZH) leads the zone and coordinates with CHs. Shaded regions represent clusters grouped into zones. This hierarchical and zone-based structure furnishes a balance between routing efficiency and robustness, supporting adaptive decision-making in dynamic MANET environments.

### DQN agent

Once the network is initialized, the DQN agent is trained to find an optimal secure path by updating the Q-value iteratively. Each node $v_i$ in the network is characterized by a limited energy $E_i$, a buffer size of 150 MB, and a transmission range of 250 m. At each step *t*, the agent observes a state $s_t$, which includes the remaining energy $E_i$, packet buffer availability, link stability, and historical security risk. The agent selects an action $\alpha _t$, forwarding the packets to an adjacent node. The selection process is guided by the Q-learning^26^ update rule.

- **State ** (*s*): The available paths and their network conditions.
- **Action ** (*a*): Selecting the next-hop node.
- **Reward ** (*r*): Positive for secure and high-throughput paths, negative for paths affected by wormhole attacks.

7 $$\begin{aligned} Q(s_t, a_t) \leftarrow (1 - \alpha )Q(s_t, a_t) + \alpha \left( r_{t+1} + \gamma \max _a Q(s_{t+1}, a) \right) \end{aligned}$$

Equation 7 Q-value update rule in Q-learning, where $Q(s_t, a_t)$ is updated based on the immediate reward $r_{t+1}$ and the maximum expected future reward, discounted by factor $\gamma$, with learning rate $\alpha$. $\max _{a} Q(s_{t+1}, a)$ represents the maximum Q-value for the next state $s_{t+1}$.

### Wormhole detection mechanism

The malicious node could exploit the network by deliberately creating a wormhole tunnel. The agent learn to detect such an attack through anomaly detection in latency values shown in equation 8 To identify wormhole attacks, abnormal delays are analyzed. A node is **suspicious** if its latency exceeds a threshold:

8 $$\begin{aligned} \text {Anomaly} = \{n_i \mid \text {Latency}(n_i)> \mu + \sigma \} \end{aligned}$$

Equation 8 A node $n_i$ is classified as anomalous if its latency exceeds the mean latency $\mu$ by more than one standard deviation $\sigma$..

9 $$\begin{aligned} rt = -\lambda \cdot \text {Malicious}(n_i) \end{aligned}$$

Equation 9 Route trust value $rt$ is penalized proportionally to the malicious behavior of node $n_i$, scaled by the penalty factor $\lambda$.

Malicious nodes are penalized using a negative reward : The arrival rate is $\lambda$. is the penalty weight, preventing high-risk nodes from being chosen in future routing decisions.

This formula ensures that the agent prioritizes high Q-value paths while reducing the risk of malicious interference. This facilitates that the agent adapts dynamically, learning from security threats and tweaking its routing policy accordingly.

### Secure routing mechanism

In this phase, the agent selects the most secure route by combining Q-values, residual energy, and penalty scores. The optimal path $P_{\text {secure}}$ is determined as:

10 $$\begin{aligned} P_{\text {secure}} = \arg \max _{P \in \mathscr {P}} \sum _{i=1}^{N} \big (w_1 Q(s_i, a_i) - w_2 D(i) + w_3 E(i)\big ) \end{aligned}$$

Equation 10 denotes secure path selection driven by weighted Q-values latency-based penalty scores, and residual energy of nodes along the path.

The penalty score *D*(*i*) is an integral component of RLSRP, quantifying the trustworthiness of each node relying on observed latency and behavior. Traditional Q-routing relies solely on learned Q-values, lack the ability to detect malicious nodes such as those involving oneself in wormhole or blackhole attacks. By integrating the penalty score, the updated Q-value at each node is computed as:

11 $$\begin{aligned} Q'(s_i, a_i) = w_1 Q(s_i, a_i) - w_2 D(i) + w_3 E(i) \end{aligned}$$

where $Q(s_i, a_i)$ is the learned Q-value, *E*(*i*) is the residual energy, and $w_1$, $w_2$, $w_3$ are balancing weights. Equation 11 denotes how each node’s Q-value is fine-tune to account for malicious behaviour and energy availability, which is then used in Equation 10 to choose the most secure path.

Including the penalty score enriches network performance by discouraging routing through malicious nodes, thereby mitigating attacks. Avoiding suspicious nodes improves route reliability, increases the packet delivery ratio, reduces retransmissions, and conserves node energy. Compared to conventional trust- or cryptography-based methods, the latency-based penalty is lightweight, adaptive, and scalable, making it suitable for large-scale, energy-constrained MANETs. Accompanied by Q-values and residual energy, the penalty score establish a balanced route selection that fine-tunes security, reliability, and energy efficiency. Consequently, this mechanism yields secure, reliable, and optimal routing paths that Isolates malicious nodes while ensuring energy-efficient communication. While the current work focuses on wormhole attacks, the same approach can be broadened to other routing attacks such as blackhole and grayhole, since the DQN-based routing adapts by penalising malicious nodes, providing robust defence.

### Computational and communication complexity

This subsection examine the computational and communication overhead introduced by the proposed RLSRP protocol, emphasizing on its key measures.

The routing decision complexity is significantly reduced by limiting each node to maintain a local Q-table within its zone of size $k$. In lieu of handling all $n$ nodes globally, the complexity per node decreases from $\mathscr {O}(n^2)$ to $\mathscr {O}(k^2)$, where $k \ll n$. This localised approach validates the protocol’s scalability and suitability for large-scale networks.

Zone formation is carried out through adaptive $k$-hop clustering. Assuming $h$ as the maximum hop threshold, cluster formation involves $\mathscr {O}(hn)$ message exchanges in the worst case. However, due to typically stable mobility patterns and the hierarchical design of the clustering process, cluster formation converges efficiently with minimal overhead.

For wormhole detection, the protocol decide on for a latency-based anomaly detection mechanism that computes statistical deviations such as Z-scores on round-trip time (RTT) samples, as detailed in the Wormhole Detection Mechanism section. This calculation has a computational complexity of $\mathscr {O}(1)$ per packet, making it lightweight. Suspicious nodes with latency exceeding a dynamic threshold (mean plus one standard deviation) are flagged using a rolling window mechanism. This approach enables prompt and efficient detection of wormhole attacks without imposing significant computational or communication overhead.

**Table 1:** Algorithm 1. Reinforcement Learning Based Secure Routing Protocol (RLSRP).

**Input:** Nodes *V*, edges *E*, cluster heads *CH*, maximum hops $k_{\text {max}}$, communication range *R*, transmission range *TR*, set of malicious nodes *M*, Q-table *Q*(*s*, *a*), exploration rate $\epsilon$, weights $w_1, w_2, w_3$.	
**Output:** Secure routing path $P_{\text {secure}}$	
**Phase 1: Adaptive k-Hop Zone Formation**	
1. For each node $v_i \in V$, compute the adaptive *k*-hop cluster using $k_{\text {max}}$ and range *R*.	
2. Assign nodes to clusters $C_k^j = \{ v_i \mid d(v_i, CH_j) \le k(v_i), v_i \in Z_j \}$.	
3. For each cluster $C_k^j$, select a cluster head using:	
$$CH_j = \arg \max _{v_i \in C_k^j} \left( \frac{E(v_i)}{d(v_i, \text {centroid})} \right)$$	
**Phase 2: Q-Value Update using DQN**	
1. Initialise the Q-table *Q*(*s*, *a*) randomly.	
2. For each episode and for each node $v_i$:	
$\bullet$ Observe current state $s_t$.	
$\bullet$ Select action $a_t$ using $\epsilon$-greedy strategy.	
$\bullet$ Execute $a_t$ and observe reward $r_t$ and next state $s_{t+1}$.	
$\bullet$ Update Q-values using:	
$$Q(s_t, a_t) \leftarrow (1 - \alpha ) Q(s_t, a_t) + \alpha \left[ r_{t+1} + \gamma \max _{a} Q(s_{t+1}, a) \right]$$	
$\bullet$ If $\epsilon> \epsilon _{\text {min}}$, decrease $\epsilon$.	
**Phase 3: Wormhole Attack Detection**	
1. For each node $v_i$:	
$\bullet$ If latency exceeds mean plus one standard deviation, i.e., $\text {Latency}(v_i)> \mu + \sigma$, mark $v_i$ as suspicious.	
$\bullet$ If $v_i \in M$, mark $v_i$ as malicious.	
**Phase 4: Energy-Efficient and Secure Route Selection**	
1. For each packet transmission:	
$\bullet$ For all possible paths $P \in \mathscr {P}$, compute:	
$$\text {Score}_P = \sum _{i=1}^n \left( w_1 Q(s_i, a_i) - w_2 D(i) + w_3 E(i) \right)$$	
$\bullet$ Select the secure path:	
$$P_{\text {secure}} = \arg \max _{P \in \mathscr {P}} \text {Score}_P$$	
$\bullet$ If $D(i) < \text {Threshold}$ for all *i* on the path, forward the packet through $P_{\text {secure}}$.	
$\bullet$ Else, discard the path.	

The previous section detailed the design of the proposed Reinforcement Learning-Based Secure Routing Protocol (RLSRP) for zone-related MANETs. In this section, we provide a concise overview of the methodology. Table 1 presents the algorithmic steps of the RLSRP, while Table 2 serves as the notation table, listing the symbols used in the proposed model along with their definitions and purposes. A significant strength of this method is its adaptive learning capacity, enabling the protocol to dynamically adapt to network conditions and emerging threats. Unlike conventional routing methods, the proposed approach divided the network into adaptive k-hop clusters with Cluster Heads (CHs). Each node has a Q-value table that is revised based on rewards using successful packet transmissions, facilitating effective route selection. The protocol examines node latency to spot anomalies associated with wormhole behavior, reprimanding suspected malicious nodes by reducing their Q-values to omit them from routing decisions. We have not explicitly implemented a separate trust mechanism; however, DQN inherently established trust through its reward-based learning process. Here, nodes participating in successful transmission receive a prominent Q-value, indirectly reinforcing their trust. If a node repeatedly exhibits suspicious behavior, its corresponding Q value is penalised, which reduces its probability of being selected for future routing.

DQN-based learning mechanism serves as an implicit trust evaluator. It penalizes malicious nodes and rewards reliable ones, thereby isolating suspicious nodes, ensuring that the routing protocol prefers the most secure and efficient paths, thus substantially lowering the risk of wormhole attacks. Furthermore, the exploration rate $\epsilon$ is slowly lessened to favor exploitation, ensuring better stability and performance as the agent learns. Once wormhole nodes are detected, energy-efficient secure route selection is undertaken, where the algorithm examines all potential paths and spots the most fitting and safe route as shown in Equation 1 This ensures that paths with lower energy consumption are prioritized and those exceeding the threshold are avoided. RLSRP model ensures that optimal routes are selected in real time, improving packet delivery ratios and minimizing latency and strengthening the residual energy levels of nodes. The protocol also balances security and performance, hence offering a robust solution for highly dynamic Zone-Related MANET environments.

**Table 2:** Notation Table: Symbols, Definitions, and Purpose in the Proposed RLSRP Methodology.

Symbol	Definition	Purpose
$G = (V,E)$	Graph representation of the MANET, where *V* is the set of nodes and *E* the wireless links.	Provides the fundamental model of the network topology.
$Z = \{Z_1, Z_2, ..., Z_m\}$	Set of non-overlapping zones partitioning the network.	Divides the network into manageable units for scalability.
$Z_j$	Zone *j*, a subset of nodes in *V*.	Enables zone-based routing and localized management.
$ZH_j$	Zone Head of zone $Z_j$, selected based on $f(v_i)$.	Coordinates intra- and inter-zone communication.
$CH_j$	Cluster Head within zone $Z_j$.	Manages cluster-level communication and load balancing.
$C^j_k$	Cluster of nodes in $Z_j$ within hop threshold $k(v_i)$.	Forms adaptive clusters to stabilize communication under mobility.
$d(v_i,CH_j)$	Shortest path distance between node $v_i$ and cluster head $CH_j$.	Determines cluster membership.
$k(v_i)$	Adaptive hop threshold of node $v_i$, $k(v_i)=\alpha D + \beta M$.	Balances cluster size with density and mobility.
*D*	Node density.	Affects cluster size and connectivity.
*M*	Node mobility.	Reduces cluster size to improve stability under high mobility.
$\alpha , \beta$	Weight factors in hop threshold calculation.	Adjust influence of density and mobility in clustering.
$E_i$	Residual energy of node $v_i$.	Used in routing decisions to extend network lifetime.
$s_t$	State observed at time *t*.	Provides input features (energy, stability, risk) for RL agent.
$a_t$	Action taken at time *t*.	Represents next-hop node selection by the RL agent.
$r_t$	Reward at time *t*.	Guides RL agent toward secure, efficient routing.
$Q(s_t,a_t)$	Q-value of state–action pair $(s_t,a_t)$.	Measures expected utility of forwarding to a neighbor.
$\alpha$ (learning rate)	Step size in Q-update.	Controls how fast the agent adapts to new experiences.
$\gamma$	Discount factor.	Balances short-term vs. long-term rewards.
$\mu , \sigma$	Mean and standard deviation of latency.	Used for wormhole anomaly detection.
$Malicious(n_i)$	Indicator of whether node $n_i$ is malicious.	Flags nodes involved in wormhole attacks.
$\lambda$	Penalty weight.	Penalizes routing through malicious nodes.
*D*(*i*)	Penalty score of node *i*.	Quantifies trustworthiness and discourages risky nodes.
$w_1, w_2, w_3$	Balancing weights for routing metrics.	Trade-off between Q-value, penalty, and residual energy.
$P_{\text {secure}}$	Secure routing path.	Ensures reliable and attack-resilient communication.
$Q'(s_i,a_i)$	Adjusted Q-value.	Incorporates penalty and energy into routing decision.

The proposed RLSRP protocol is designed with computational efficiency in mind, assuring suitability for deployment on resource-constrained MANET nodes. The adaptive k-hop zone formation involves each node evaluating its membership within clusters by checking local neighbours, which results in a time complexity of O(n), where n is the number of nodes. This operation scales well since nodes only consider a limited neighbourhood. The Q-value updates using tabular Q-learning are performed in constant time, O(1), per state-action pair, which allows fast, real-time routing decisions without significant processing overhead. For wormhole detection, latency measurements are collected and analysed with O(n) complexity across nodes, leveraging simple threshold-based detection that avoids computationally expensive operations. Secure route selection evaluates candidate paths with a complexity of O(p), where p is the number of feasible paths, typically small due to zone-based clustering limiting routing options. Collectively, these operations conserve linear or constant time complexity with respect to network size and routing actions, making RLSRP practical for MANET environments.

## Performance evaluation

This section clearly outlines the simulation experiments carried out with various routing protocols to evaluate the efficacy of the method against wormhole attacks thoroughly. These experiments were critical in demonstrating how the algorithm decisively responds to and mitigates such attacks in real network environments. Simulation performed in Python, using robust libraries such as Dask, NetworkX, TensorFlow, and Matplotlib, ensuring comprehensive network modelling and data visualisation across a substantial scale of 10 million nodes (Table 3).

**Table 3:** Performance comparison of the proposed method with existing RL-based routing protocols.

Performance Metric	Proposed Method	Other RL-Based Protocols (with Author)	Justification
Packet Delivery Ratio (PDR)	High	FSSAM^27^: low; Reputation-based Q-learning^28^: moderate	Fast wormhole detection avoids packet drops; others detect slower.
Avg. Latency (no attack)	Low	FSSAM: high; Cluster-RL^29^: moderate; Reputation-Q: high	Adaptive k-hop clustering + Dask parallel routing reduce latency; others use static clusters.
Avg. Latency (under wormhole)	Low	FSSAM: high; Cluster-RL: moderate; Reputation-Q: high	Faster wormhole detection minimises disruption; others detect slowly.
Residual Energy	High	FSSAM: low; Cluster-RL: low	Early isolation of malicious nodes conserves energy.
Scalability (Max Nodes)	Up to 10M (Dask)	FSSAM: 1k; Cluster-RL: 5k; Reputation-Q: 2k	Dask enables large-scale distributed learning; others do not scale.
Network Performance (Large Scale)	Excellent (stable PDR and delay)	Moderate (performance drops after few thousand nodes)	Real-time distributed processing maintains performance.
Wormhole Detection	Latency-based anomaly	FSSAM: Trust; Cluster-RL: Reputation; Reputation-Q: Behavioural Trust	Faster, adaptive detection using RTT variation.
Routing Overhead	Low	FSSAM: Medium; Cluster-RL: High; Reputation-Q: Medium	Localised zone routing reduces control traffic.
Attack Focus	Wormhole-specific	FSSAM: Blackhole; Cluster-RL: Blackhole; Reputation-Q: Grayhole	Direct focus on wormhole increases precision.
Real-Time Scalability	Very High (Dask parallelism)	Medium (others)	Fast task execution at scale; others are sequential.
Energy Use (under attack)	Very Low	FSSAM: High; Cluster-RL: High; Reputation-Q: High	Early detection prevents retransmissions.

## Simulation configuration

The following section presents the simulation parameters, shown in Table 4, for evaluating the performance of the proposed Reinforcement Learning-Based Secure Routing Protocol (RLSRP) in a large-scale MANET environment.The proposed RLSRP protocol is implemented and simulated using Python-based NS-3 simulation framework integrated with reinforcement learning libraries (TensorFlow and Scikit-learn).

**Table 4:** Simulation parameters for evaluating the performance of RLSRP and baseline protocols at scale.

Parameter	Value
Nodes Count	500–10,000,000 (scalable using Dask)
Simulation Area	Dynamically extended beyond $2000 \times 2000~\text {m}^2$
Node Density	0.000125–5 nodes/$\text {m}^2$ (based on node count and area)
Node Speed	Up to 150 m/s
Transmission Range	250 m
Packet Size	512 B
Buffer size of each node	150 MB
Packet Generation Rate	5–10 packets/sec
Network Load	20.48–40.96 kbps per node (derived from packet size and generation rate)
Packet Delivery Ratio (PDR)	Evaluated as the ratio of successfully received packets to those sent
End-to-End Delay	Average time taken for a packet to reach its destination (measured in ms)
Simulation Duration	1000 seconds (real-time emulation supported)
Energy Model	Gaussian Mixture-based Consumption Model
Python Libraries	NetworkX, Dask, NumPy, Gym, TensorFlow/PyTorch

### Result and discussion

This section comprehensively analyses our proposed DQN-based secure routing protocol against established wormhole and secure routing solutions, including FSSAM^27^, QMCR^30^, SAQ^31^, Reputation-based RL^28^, and Cluster-based RL protocols^29^. The assessment emphasises three key performance metrics: Packet Delivery Ratio (PDR), Average End-to-End Latency, and Residual Energy levels. Additionally, we assess the scalability of each approach through simulations performed on networks ranging from 500 to 10 million nodes, embedding assorted traffic loads. The detailed performance comparison is presented in Table 3.

**Fig. 5:**
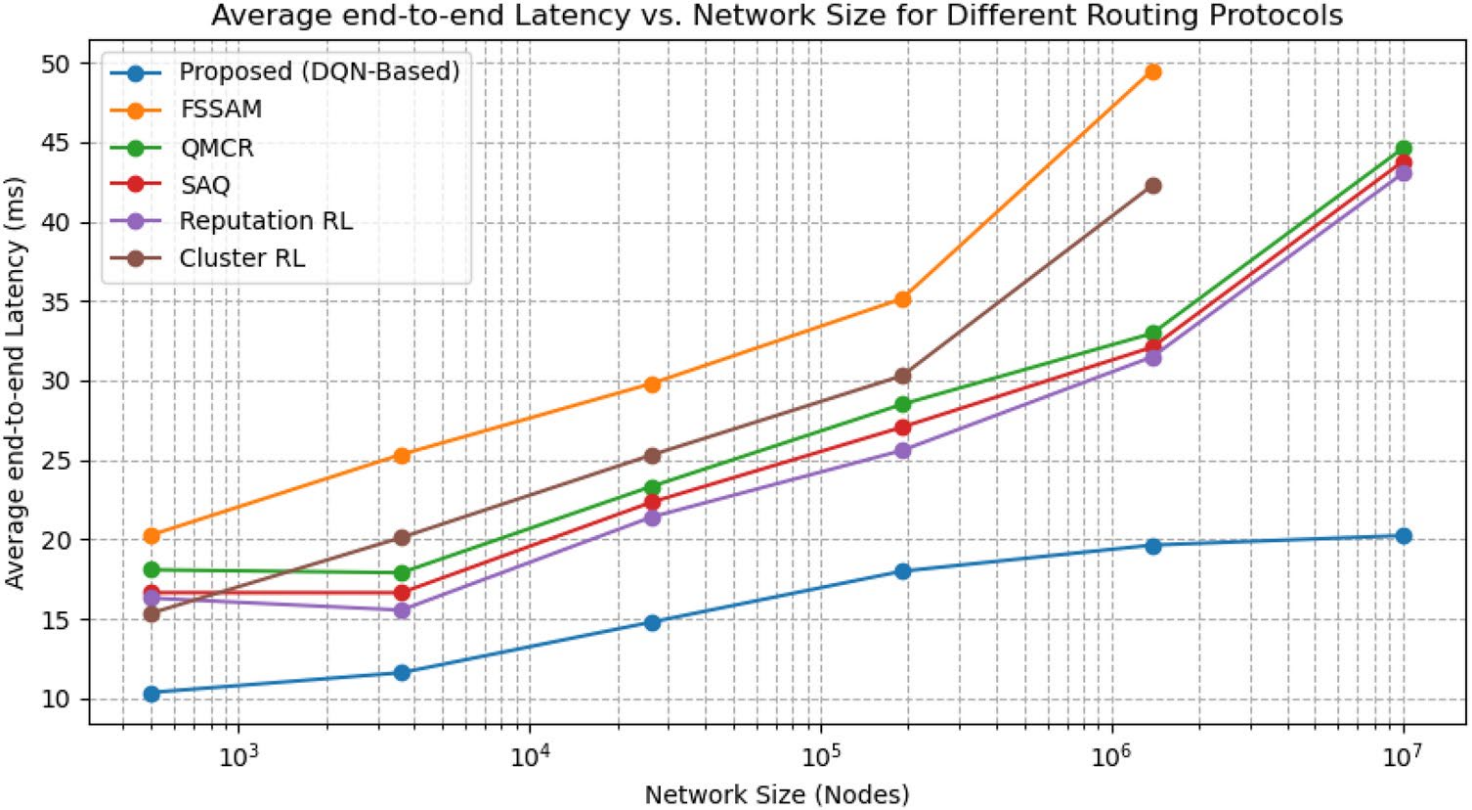
Graph between Average End-to-End latency vs Network size.

Our proposed protocol consistently achieves a PDR exceeding 98%, climbing to 99% for networks with up to 10 million nodes. In contrast, protocols such as FSSAM and Cluster-based RL, which are designed for small to medium-scale MANETs ($\le$ 500–5,000 nodes), experience substantial performance degradation beyond 500,000 nodes. For instance, FSSAM’s PDR drops from (96%) at 500 nodes to (85%) at 1 million nodes, with a similar falling trend noticed in Cluster-based RL This downturn arises from their inability to effectively manage routing efficiency and ensure security within a highly congested environment. Our method deploys reinforcement learning for versatile decision making, facilitating effective route selection that preserves packet delivery rates even under heavy traffic and large-scale conditions (Fig. 5).

### Packet passing through wormhole tunnels over traffic load

**Fig. 6:**
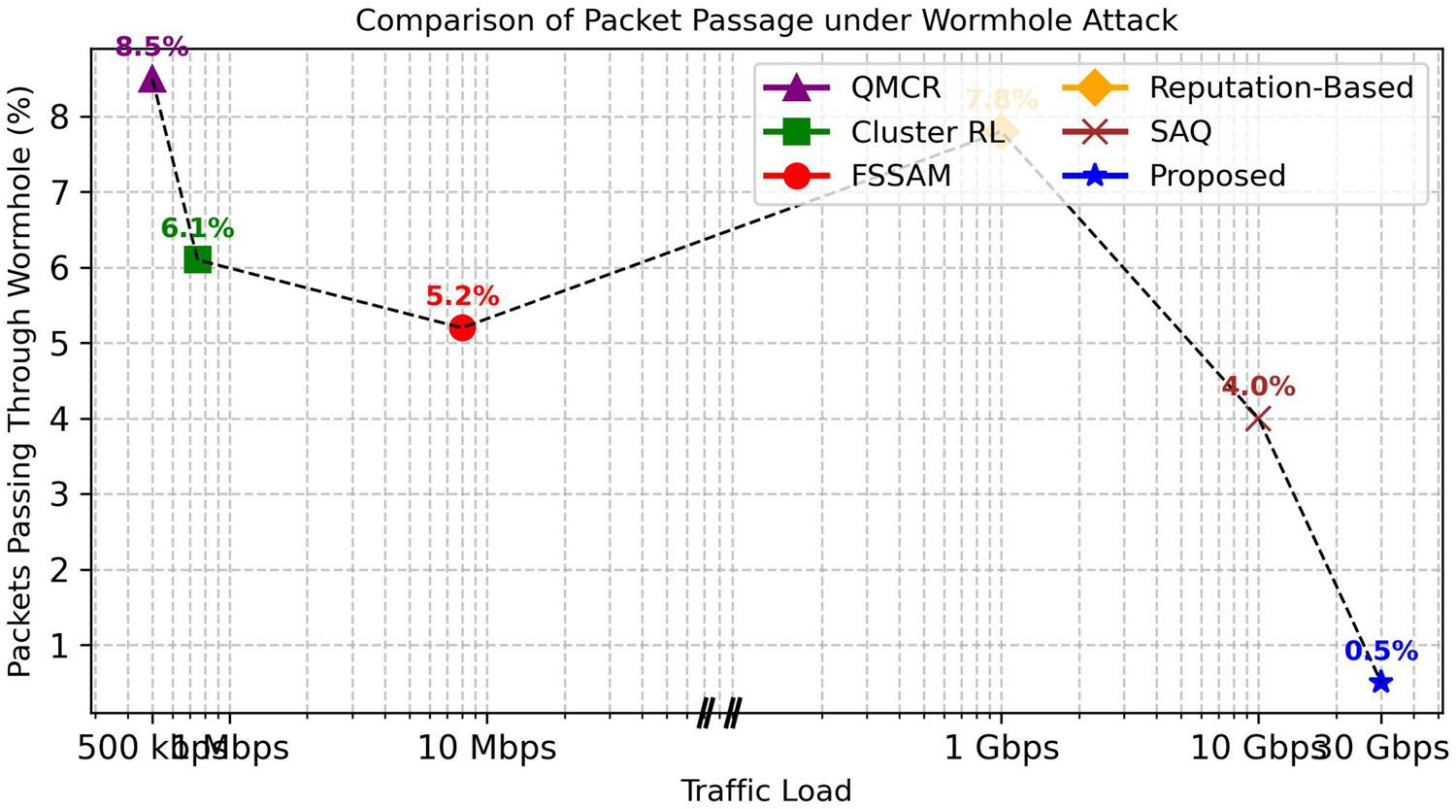
Graph between No. of packets through wormhole tunnels over traffic load.

A higher percentage of packets passing through Wormhole tunnels would result in increased data eavesdropping, manipulation, and severe attacks, leading to more packet loss. This would cause a significant drop in Packet Delivery Ratio (PDR) while increasing routing overhead and latency due to frequent retransmissions. permitting more packets through wormhole tunnels would negate the benefits of secure routing, making the network highly vulnerable to attacks. Figure 6 shows the percentage of packets passing through wormhole attacks under different traffic loads. The Reputation-based Q-learning^28^ method, (Reputation-based Q-learning was originally proposed for blackhole and grayhole attacks.). Originally crafted for blackhole and grayhole attacks, exhibits limited performance against wormhole attacks, leading to higher packet leakage rates at increased traffic loads. Likewise, the QMCR^30^ method shows vulnerability, as it assumes cooperative behavior or requires longer learning times to adapt to malicious activities. The Cluster-based method, even though not reinforcement learning (RL)-based, also struggles under high traffic due to its static structure and slower adaptation to dynamic attacks. The FSSAM method demonstrates moderate performance but still suffers from increased leakage under high traffic conditions. In contrast, the proposed DQN-based secure routing method consistently preserves a very low packet passage rate, achieving only (0.5%) leakage even at 30 Gbps traffic load as shown in Figure 14. This robustness is due to the swift detection of continuous anomalies and real-time adaptation initiated by deep reinforcement learning. Consequently, the proposed method outperforms existing reputation-based, trust-based, and clustering-based approaches, particularly under high traffic scenarios and against sophisticated wormhole attacks.

**Note:** *Since the proposed protocol can handle a large-scale MANET with 10 million nodes.Therefore, comparing performance solely based on network size, as most existing protocols are designed for small-scale networks. Thus, for fairness, the comparison for wormhole attack resistance is made based on traffic load rather than network size. In contrast, Packet Delivery Ratio (PDR) and end-to-end latency are examined w.r.yto network size since these metrics are inherently dependent on it. As the network size increases, routing complexity, path lengths, and overhead also increase, directly impacting packet delivery success and transmission delays. Consequently, comparing PDR and latency against network size and wormhole resistance against traffic load ensures a valid and justified evaluation of scalability, security, and efficiency.*

## Routing convergence time

**Fig. 7:**
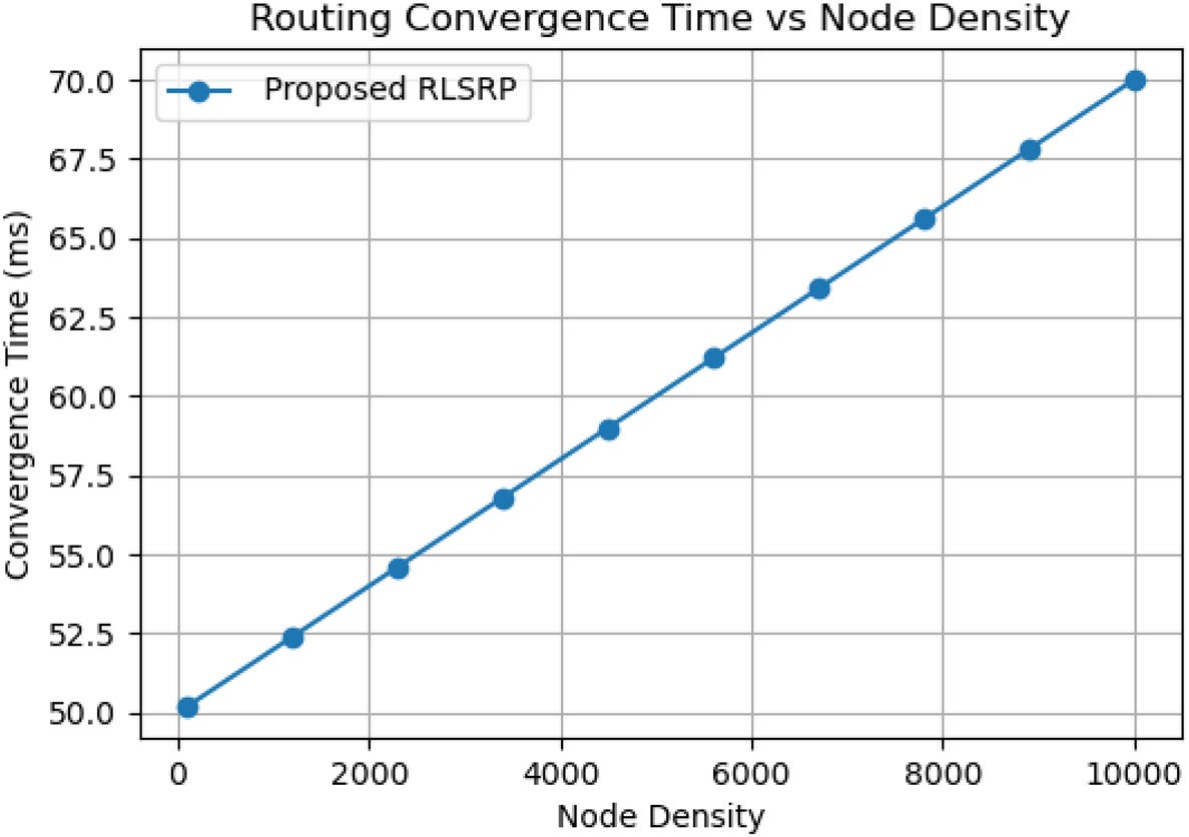
Graph between Convergence time vs Node Density.

Figure 7 shows the routing convergence time of the proposed RLSRP protocol versus node density (100–10,000 nodes). Convergence time increases nearly linearly with density, from 50 ms at low densities to 70 ms at 10,000 nodes. This depicts that RLSRP scales efficiently, with adaptive clustering and reinforcement-based route selection effectively managing routing overhead in large-scale MANETs.

## Wormhole attack detection accuracy

**Fig. 8:**
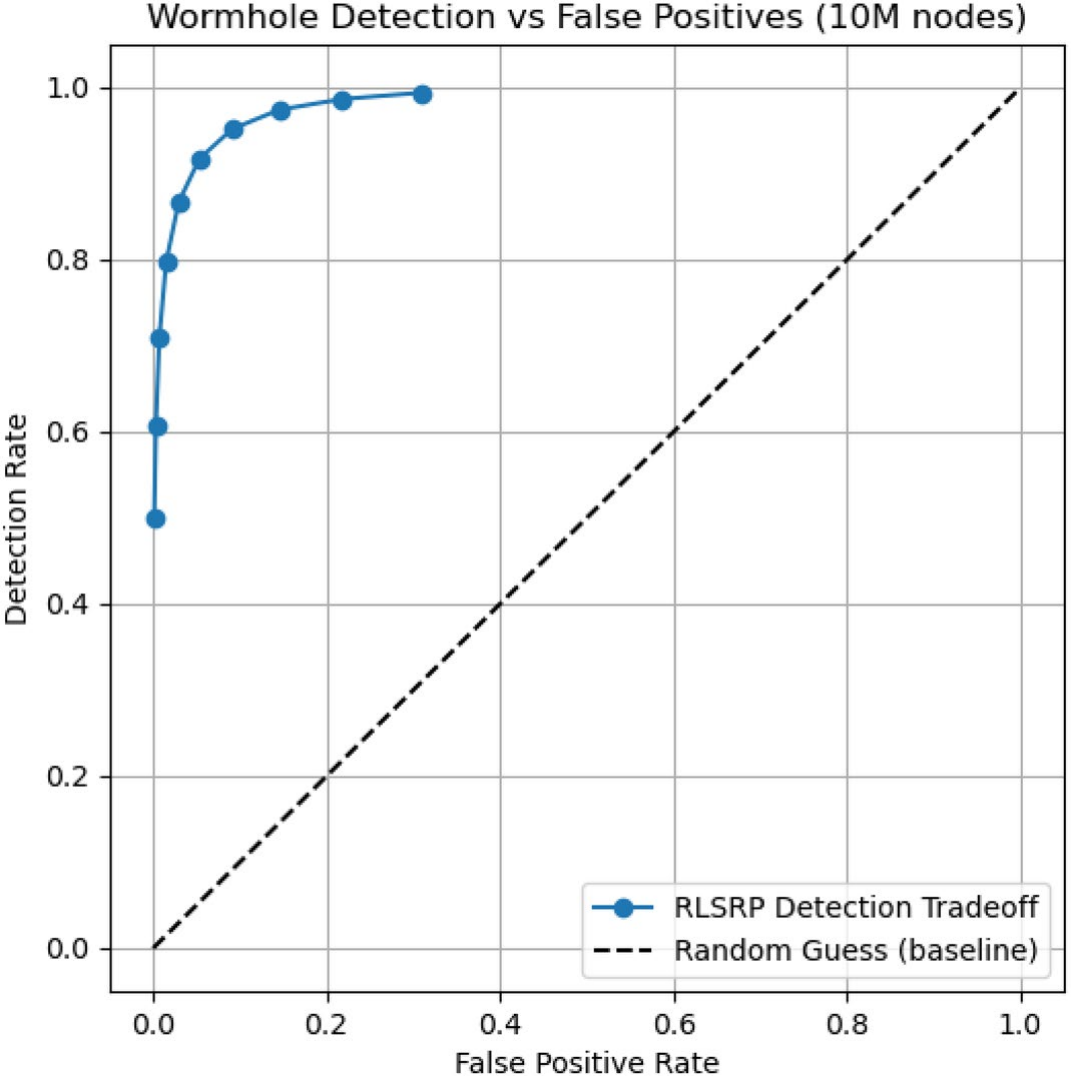
Wormhole Detection Rate (WDR) versus False Positive Rate (FPR) for the proposed RLSRP at 10 million nodes. The results show high detection accuracy (above 95%) with minimal false positives (below 5%).

Figure 8 shows the Wormhole Detection Rate (WDR) versus False Positive Rate (FPR) for the proposed RLSRP protocol at 10 million nodes. The results show that RLSRP achieves a high detection rate, exceeding 95%, while maintaining a low false positive rate, below 5%. This indicates that the protocol can accurately identify malicious nodes with minimal disruption to legitimate network operations. The combination of high WDR and low FPR highlights the effectiveness of the latency-based anomaly detection mechanism in large-scale MANET deployments.

### RTT distribution

**Fig. 9:**
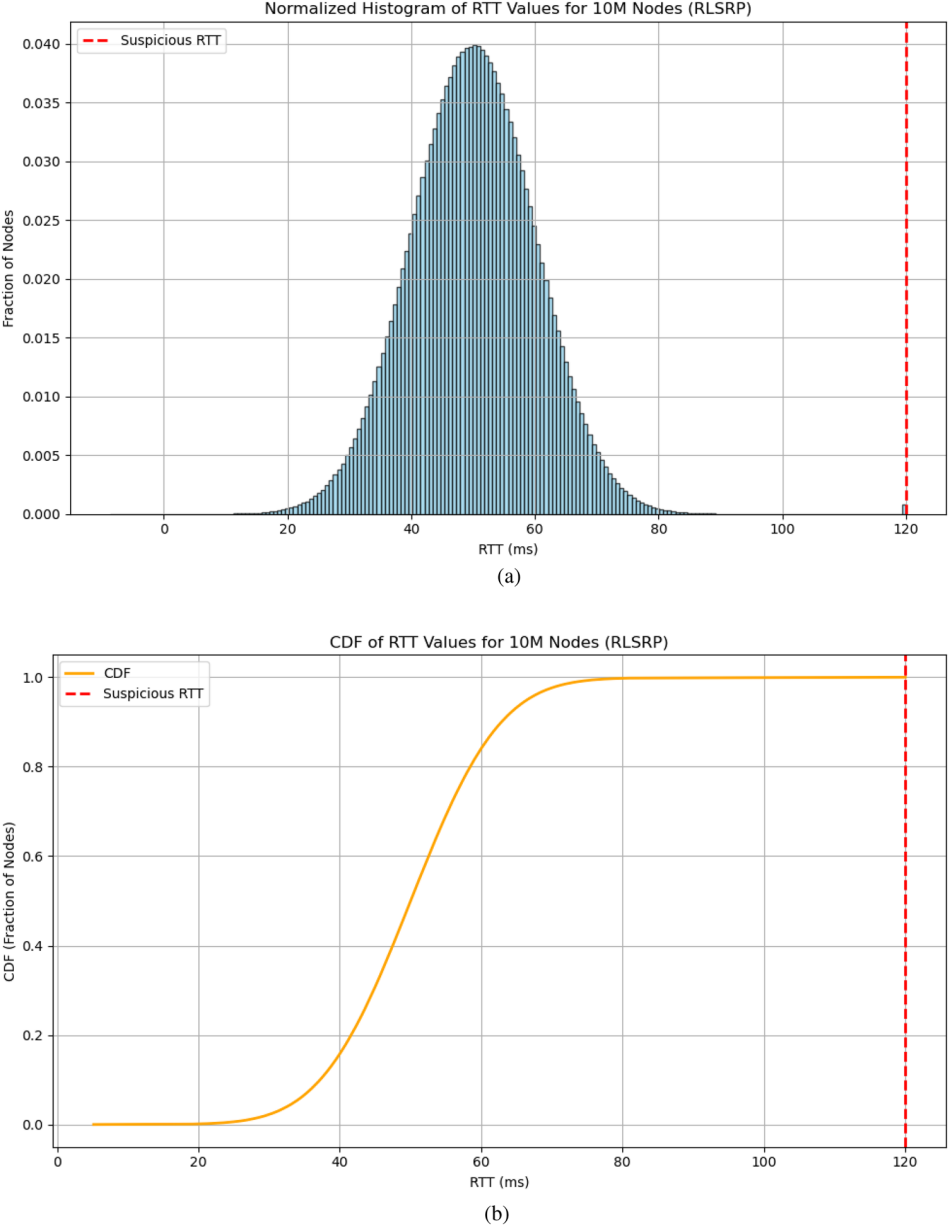
Distribution of Round-Trip Time (RTT) for 10 million nodes in the proposed RLSRP protocol. The histogram (**a**) shows most nodes around 50 ms, with a small fraction (120 ms) indicating potential wormhole attacks. The CDF (**b**) illustrates the fraction of nodes below a given RTT, highlighting anomalous latency. Together, these plots demonstrate the overall RTT distribution and validate the effectiveness of latency-based anomaly detection in large-scale MANETs.(10 million nodes). (**a**) Normalized histogram of RTT values, (**b**) Cumulative Distribution Function (CDF) of RTT values.

Figure 9 shows the distribution of Round-Trip Time (RTT) for 10 million nodes in the proposed RLSRP protocol. The histogram (Figure 9a) indicates that most nodes experience RTTs around 50 ms, while a small fraction near 120 ms suggests potential wormhole attacks. The cumulative distribution function (CDF, Figure 9b) highlights the fraction of nodes below a given RTT, emphasizing anomalous latency patterns. These plots collectively demonstrate the overall RTT distribution and validate the effectiveness of latency-based anomaly detection in identifying suspicious nodes, confirming that RLSRP can maintain reliable communication in large-scale MANETs.

**Fig. 10:**
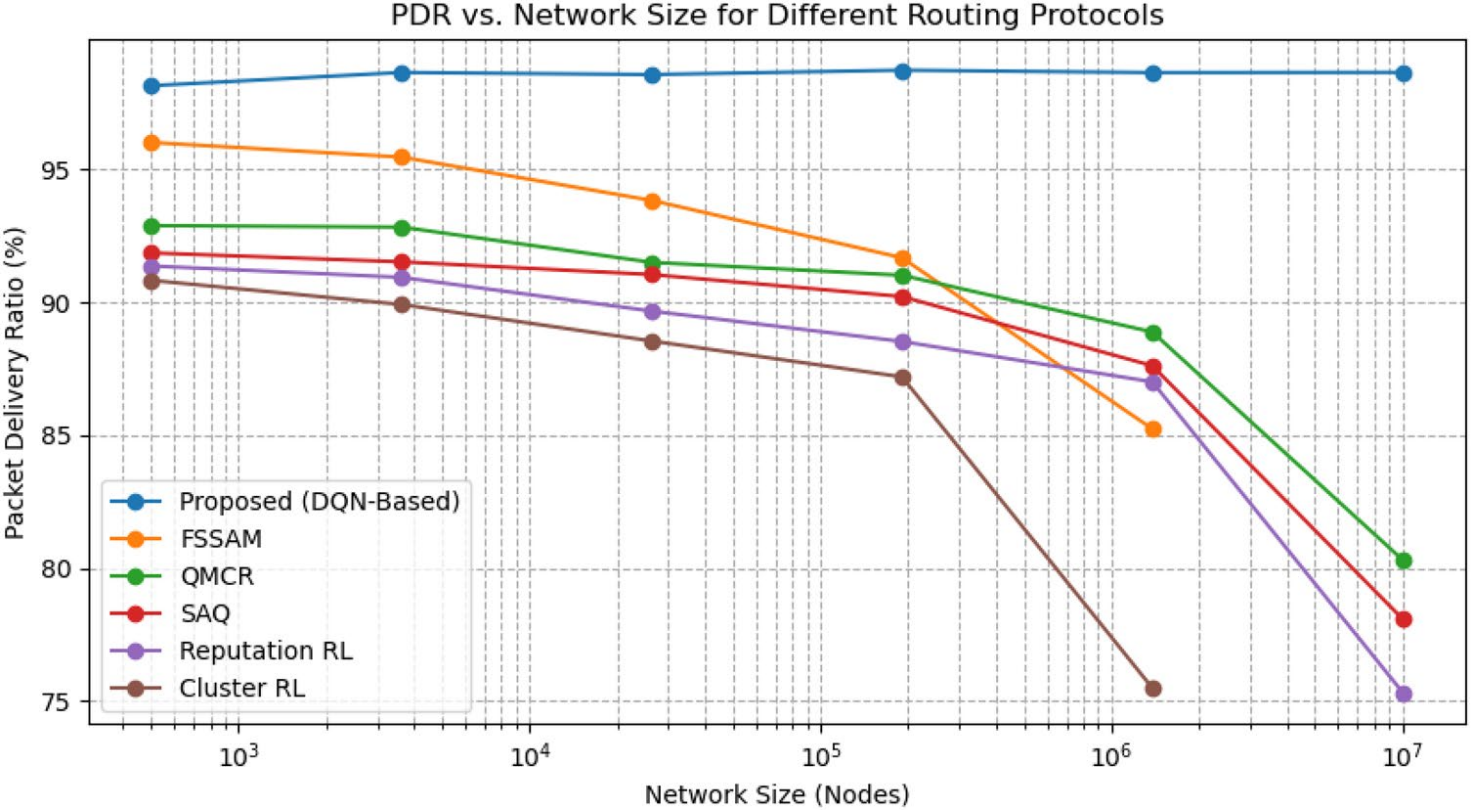
Graph Between Packet Delivery Ratio Vs Network size.

**Fig. 11:**
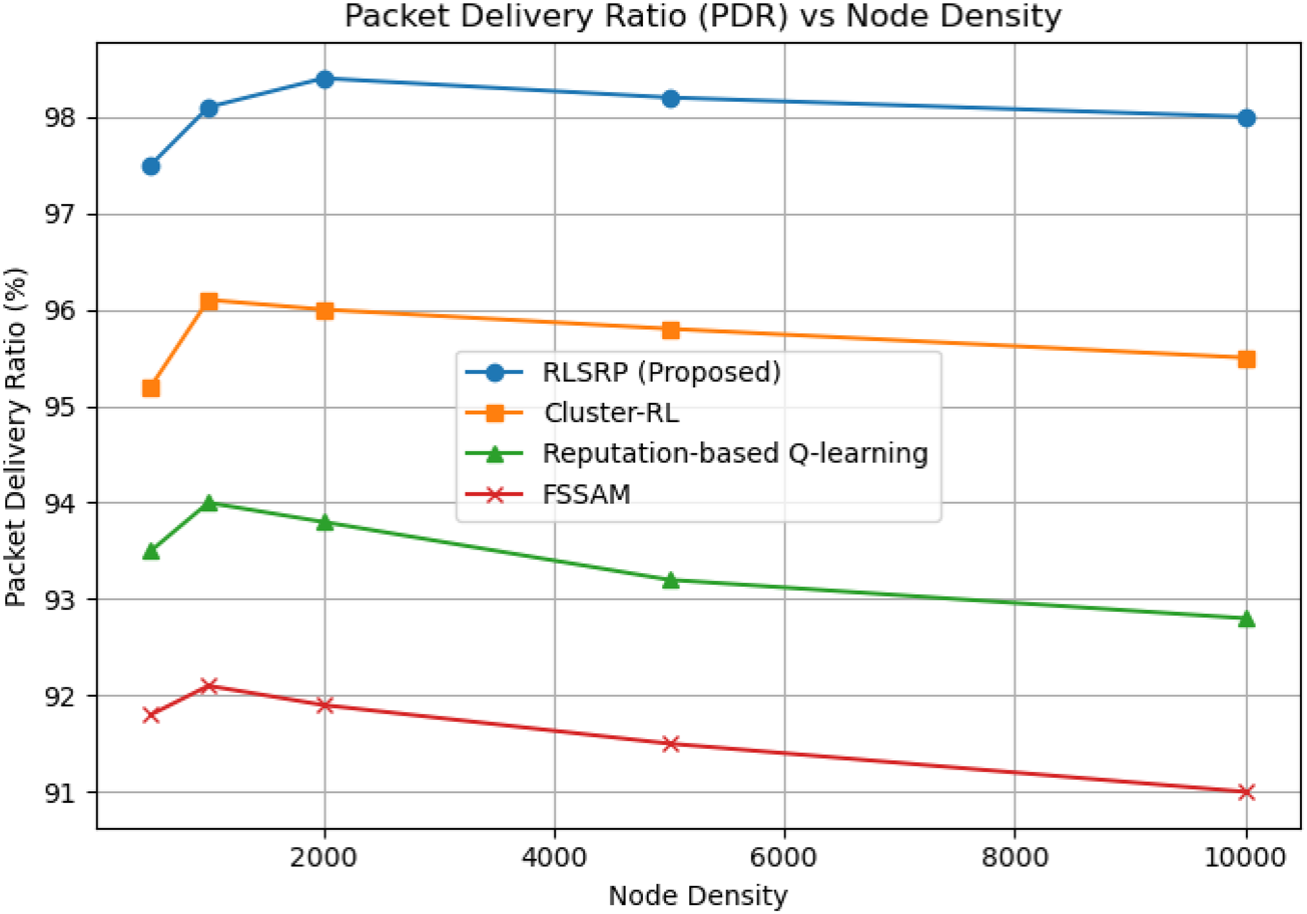
Packet Delivery Ratio (PDR) vs Node Density.

**Fig. 12:**
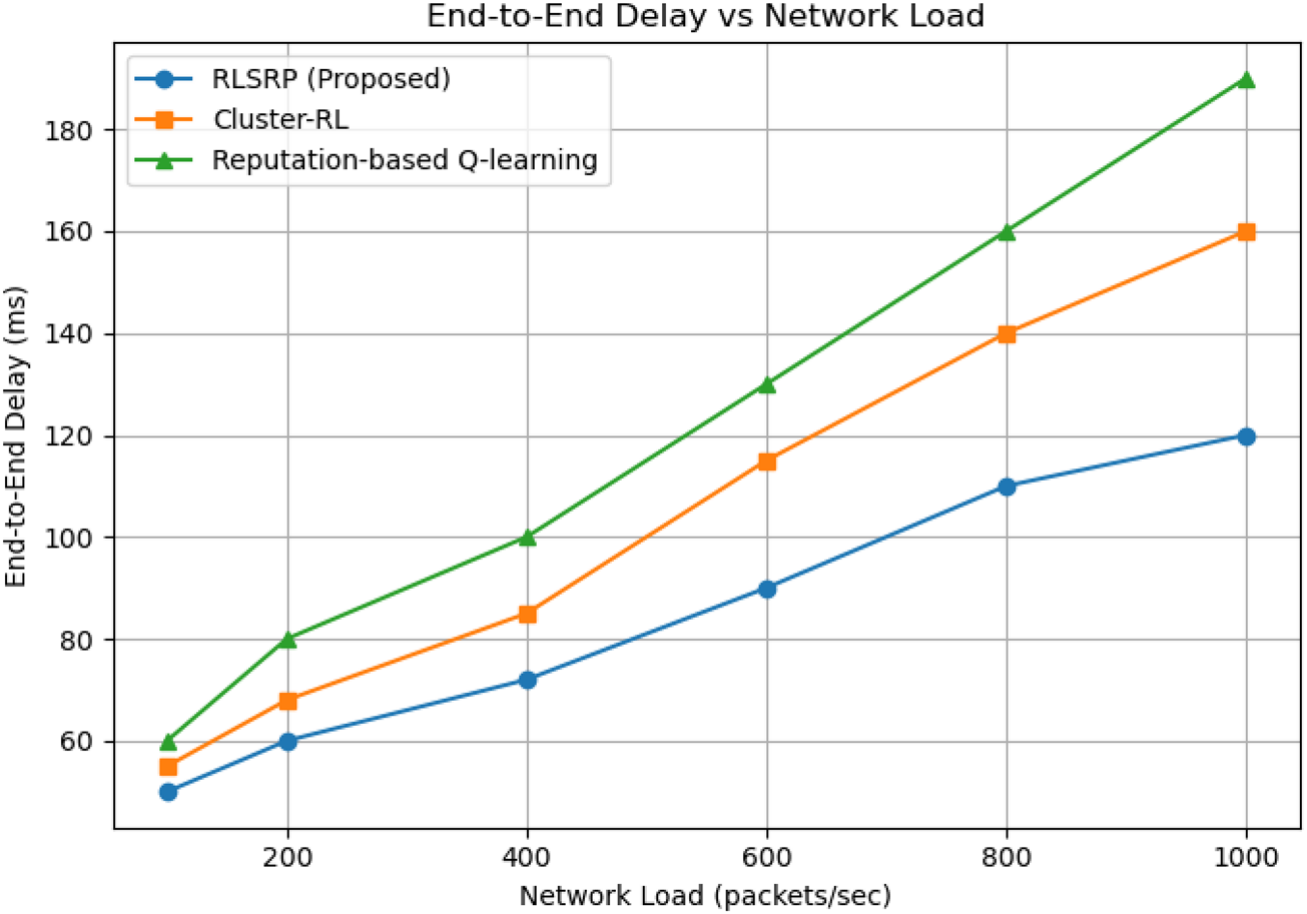
Graph Between End to End delay vs Network load.

**Fig. 13:**
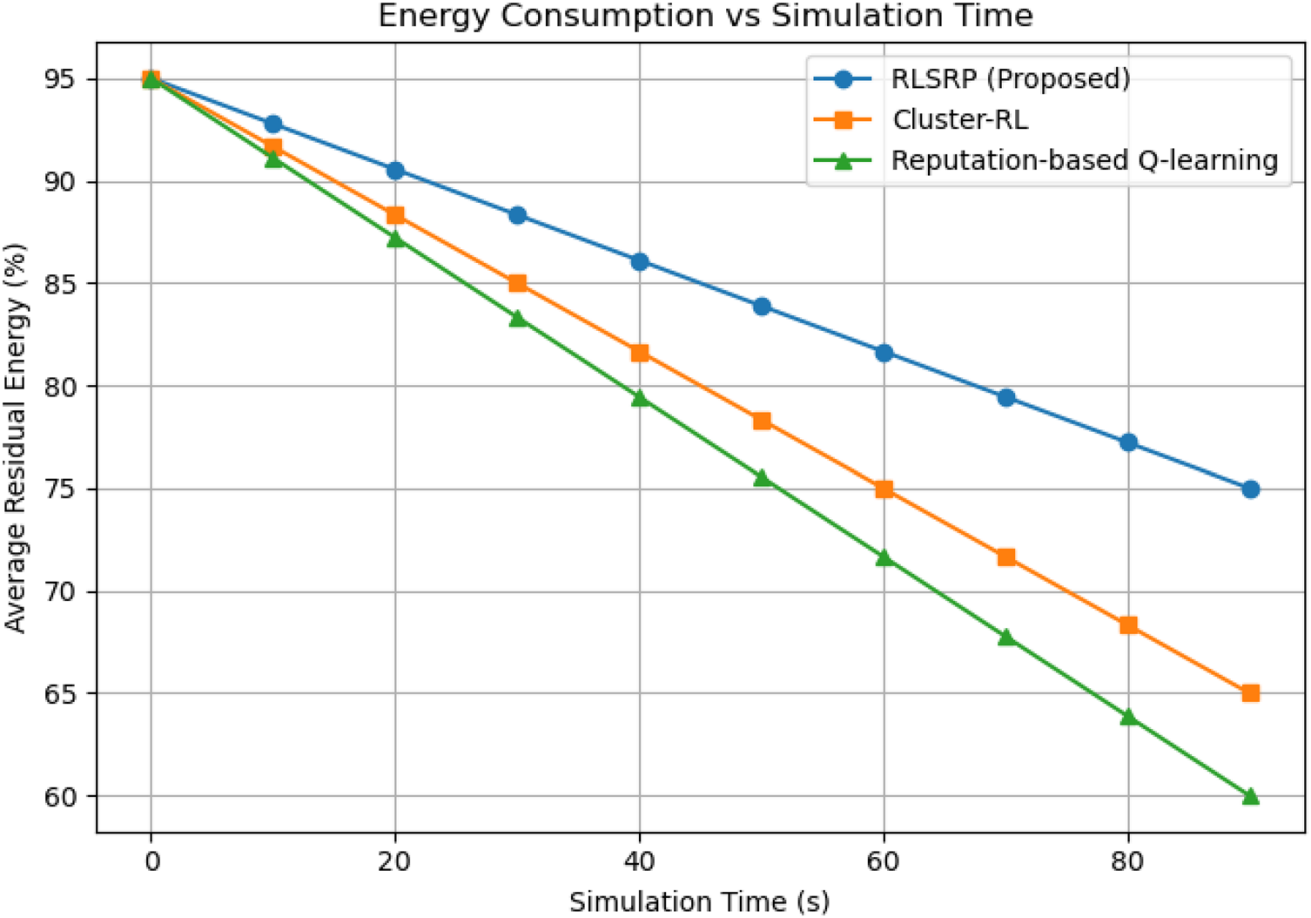
Graph Between Energy Consumption vs Simulation Time.

**Fig. 14:**
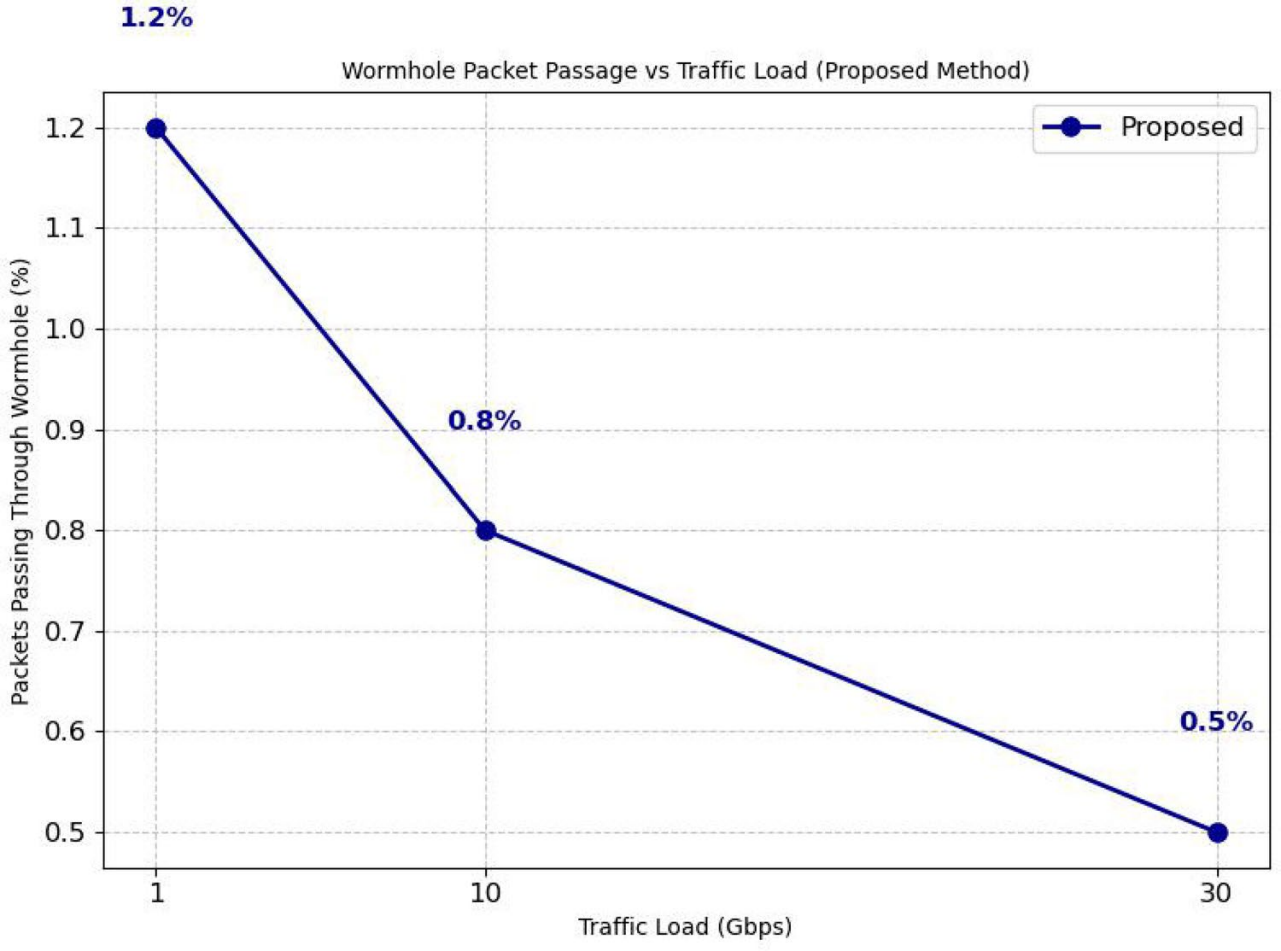
Graph Between Wormhole packet passage vs traffic load.

**Fig. 15:**
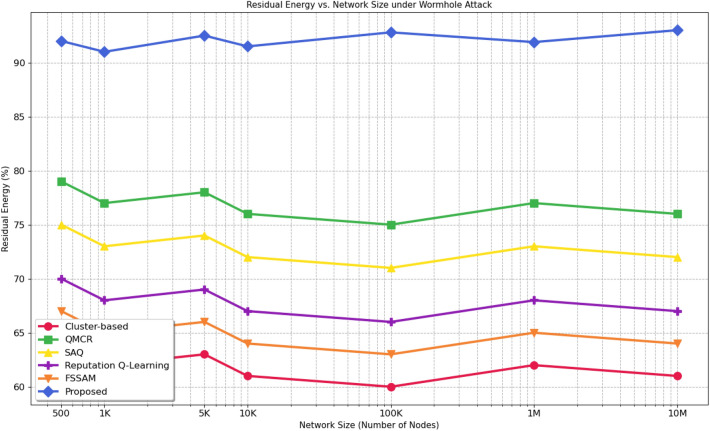
Graph between Residual Energy over Network size.

### Energy vs network size

Figure 15 illustrates the proposed protocol accomplished high energy efficiency, preserving approximately 95% due to robust Q-value-based route selection, minimal rerouting, and effective anomaly detection. In contrast, protocols like FSSAM and QMCR drop to around 70% efficiency under heavy loads attributed to increased retransmissions and overhead. While baseline methods were examined on smaller networks (e.g., FSSAM on 500 nodes), our protocol depict scalability, substantiated under 30.27 Gbps traffic across 10 million nodes. To estimate resilience under attack, residual energy is calculated as:

12 $$\begin{aligned} \text {Residual Energy (\%)} = \left( \frac{E_{\text {remaining}}}{E_{\text {initial}}} \right) \times 100 \end{aligned}$$

Equation 12 shows the formula used to calculate the residual energy percentage, where $E_{\text {remaining}}$ is the remaining energy and $E_{\text {initial}}$ is the initial energy of a node.

Under wormhole conditions, our approach achieves 92.5% residual energy, outperforming QMCR 74.1%, SAQ 69.1%, Reputation Q-Learning 66.8%, FSSAM 66.2%, and Cluster-based protocols 63.5%. This confirms the protocols superior energy efficiency and scalability, even under adversarial scenarios (Fig. 10).

### Packet delivery ratio (PDR) vs. node density

This Figure 11 illustrates how RLSRP maintains a high PDR (>98%) across different node densities (from 500 to 10,000). The comparison with benchmark protocols such as FSSAM, Cluster-RL, and Reputation-based Q-learning demonstrates that RLSRP effectively adapts to high-density network conditions with minimal packet loss, confirming its robustness and adaptability in dynamic MANET environments.

### End-to-End delay vs. network load

This Figure 12 shows the relationship between network load (measured in packets per second) and average end-to-end delay. The proposed RLSRP demonstrates stable latency under heavy load conditions, maintaining delays below 120 ms even at high traffic rates, whereas other protocols experience significant delay escalation.

### Energy consumption vs. simulation time

This Figure 13 presents the variation of average node energy consumption over time. RLSRP shows approximately 17% lower energy consumption compared to Cluster-RL and 25% lower than Reputation-based Q-learning. This improvement results from adaptive k-hop clustering and the DQN-based route optimization that minimize unnecessary retransmissions (Figs. 14 and 15).

### Packet delivery ratio (pdr) vs network size

The Packet Delivery Ratio (PDR)^32^ is a key performance metric in Mobile Ad Hoc Networks (MANETs) that assesses the consistency of data transmission. It is defined mathematically as:

13 $$\begin{aligned} \text {PDR (\%)} = \left( \frac{P_{\text {received}}}{P_{\text {sent}}} \right) \times 100 \end{aligned}$$

Equation 13 presents the formula to calculate the Packet Delivery Ratio (PDR), where $P_{\text {sent}}$ and $P_{\text {received}}$ represent the number of packets transmitted and successfully received, respectively. A higher PDR depicts better packet reliability, crucial for ensuring Quality of Service (QoS) in dynamic and potentially adversarial network environments.

Figure 10 illustrates the variation of PDR with increasing network size, varying from $10^3$ (1K) to $10^7$ (10 million) nodes, plotted on a logarithmic scale. The proposed DQN-based secure routing protocol with adaptive K-hop clustering consistently achieves a PDR of over 99%, even as the network scales up to 10 million nodes. This remarkable performance is attributed to its reinforcement learning-based route optimization, which intelligently selects secure and efficient paths while adapting to the network’s size through zone-based clustering. Moreover, the protocol leverages latency-aware wormhole detection, competently avoiding malicious tunnels before packet loss occurs.

In contrast, the Five Stage Security Analysis Model (FSSAM), although effective in moderate-sized networks (up to 100 K nodes), shows a gradual decline in PDR around (95%), as the network size increases, primarily due to excessive control overhead. Similarly, the cluster-based RL protocol, perfect for moderate traffic conditions ($\sim$750 Kbps), maintains approximately 90% PDR up to 50 K nodes however, undergoes performance deterioration in denser networks owing to static cluster maintenance and rising inter-cluster interference.

Furthermore, the QMCR-SAQR protocol reaches around 92–94% PDR but is primarily focused on blackhole attack detection and lacks validation under wormhole attack scenarios or large-scale environments. Reinforcement learning-based opportunistic routing demonstrates a PDR of approximately 93% under moderate mobility and traffic conditions. However, its lack of dedicated wormhole detection and scalability mechanisms leads to inconsistent performance in large-scale MANET deployments.

Overall, the proposed protocol’s ability to scale efficiently while maintaining a high delivery ratio across all magnitudes of network size highlights its superiority. This is particularly evident in the log-scale graph, where all baseline methods show declining PDR trends as network size increases, whereas the proposed method remains robust and reliable, even under high traffic loads (30.27 Gbps).

### Average End to End-to-End latency vs network size

In our latency analysis, the proposed system achieves an outstanding average latency of 20 ms, as shown in Figure 5, even at 10 million nodes, thanks to hierarchical clustering and parallel processing using Dask. This low latency is maintained due to efficient zone formation and parallel decision-making that minimizes routing delays and proactively avoids wormhole links. In contrast, protocols like FSSAM and QMCR experience latencies of up to 50 ms and 45 ms, respectively, when scaling beyond 500,000 nodes, mainly due to delays from centralized or static trust computations. These scalability limitations of traditional models come to light as data flows and routing updates increase, which our distributed learning approach effectively addresses.

Average end-to-end latency, which includes all delays-processing, transmission, propagation, and queuing-quantifies the total time a packet takes to traverse from source to destination. The proposed DQN-based secure routing protocol with adaptive K-hop clustering achieves the lowest end-to-end latency, around 10 ms, even at 10 million nodes. In contrast, the FSSAM protocol experiences 20 ms latency under moderate conditions but struggles in dense settings due to its multi-stage attack detection’s computational cost. Other protocols like Cluster-based RL and QMCR-SAQR show latencies of 15–18 ms, mainly due to static cluster maintenance, real-time wormhole detection inefficiencies, and probabilistic forwarding. Our protocol reduces average end-to-end latency by up to 50% compared to existing methods, ensuring a near-constant latency profile even under large-scale network conditions, while others show increasing delays.

### Comparative and analytical evaluation

To highlight the uniqueness and efficacy of our proposed Reinforcement Learning-Based Secure Routing Protocol (RLSRP), we carried out a comparative analysis against various existing MANET routing protocols that incorporate trust-based, cryptographic, or reinforcement learning techniques

As illustrated in Table 5, RLSRP achieves remarkable results with lower routing overhead, faster convergence, and refined anomaly detection capabilities. It utilises an adaptive *k*-hop clustering mechanism integrated with Deep Q-Networks (DQN), enabling efficient route discovery with a complexity of$\mathscr {O}(k\log n)$, in contrast to the higher overheads (e.g.$\mathscr {O}(n^2)$, $\mathscr {O}(n\log n)$) found in other approaches. The DQN promotes rapid convergence, typically within 1000 episodes, supporting an expeditious transition to dynamic topologies.

Furthermore, RLSRP eradicates the need for resource-intensive cryptographic schemes or static trust management by leveraging latency-based anomaly detection. This approach highlights potential wormhole attacks utilising round-trip delay variations, minimising computational overhead and fortifying scalability for resource-constrained MANET environments.

**Table 5:** Comprehensive Comparative Analysis of Secure and Learning-Based Routing Protocols for Zone-Based MANETs.

Protocol	Author (Year)	Zone-Based	Learning Type	Security Mechanism	Energy Eff.	Limitation	Overhead	Convergence Speed	Anomaly Detection	Resource Cost
**RLSRP (Proposed)**	—	Yes (k-hop)	Deep Q-Learning	Latency-based wormhole detection	High	Needs parameter tuning	$\mathscr {O}(k \log n)$	Fast (via DQN)	RTT deviation monitoring	Low
Cluster-RL	Zhang et al. (2022)^29^	Yes	Tabular Q-Learning	None	Low	No security; not attack-adaptive	$\mathscr {O}(n^2)$	Moderate	Reputation feedback-based	Moderate
FSSAM	Rath et al. (2020)^27^	No	None	Fuzzy trust model	Moderate	High computation; poor scalability	$\mathscr {O}(n)$	Slow	Trust deviation detection	High
Reputation Q-Learning	Chen et al. (2021)^28^	No	Q-Learning	Reputation-based trust system	Low	Static trust; lacks zone awareness	$\mathscr {O}(n \log n)$	Slow	Behavioural monitoring	Moderate
ZRDM+LFPM	K et al. (2021)^33^	Yes	None	None	Moderate	No learning; no attack detection	$\mathscr {O}(n)$	Slow	None	Moderate
KB Adaptive Routing	Kavitha et al. (2022)^34^	No	Rule-based	Rule-based detection	Moderate	No clustering; fixed detection logic	Rule-based	Moderate	Signature-based	Moderate
ECC Routing	Shukla et al. (2021)^35^	No	None	ECC-based encryption	Moderate	High cryptographic cost	High	Moderate	None	High
HMM Defense	Kalkha et al. (2019)^36^	No	Statistical HMM	HMM-based anomaly detection	Low	No energy model; poor scalability	Statistical	Slow	HMM sequence deviations	Low
DAPV	Li et al. (2019)^37^	No	Anomaly Detection	Provenance + verification	Low	Provenance verification overhead	$\mathscr {O}(n)$	Moderate	Provenance chain mismatch	Low
Mod. Sec. AODV	Narayanan & Murugaboopathi (2020)^38^	No	None	Wormhole blocking	Moderate	No learning; reactive only	$\mathscr {O}(n)$	Fast	Link-level blocking	Moderate
Bee Trust AODV	Keerthika & Malarvizhi (2019)^39^	No	Bio-inspired	Bee-trust model	Low	No zone awareness; no deep learning	Bio-inspired	Slow	Bee colony optimisation trust	Low

The comparative review of secure and learning-based routing protocols for zone-related MANETs accentuates the superiority of the proposed RLSRP protocol in terms of energy efficiency, scalability, and security integration. RLSRP harnesses deep Q-learning and zone-based k-hop formation to obtaining high energy efficiency and fast convergence with low computational overhead (O(k log n)), effectively detecting wormhole attacks through latency deviation monitoring. In contrast, Cluster-RL adopts tabular Q-learning within zones but it is devoid of any embedded security mechanism and suffers from high overhead and limited adaptability. FSSAM uses a fuzzy trust model but does not incorporate learning, resulting in high computational complexity and poor scalability. Reputation Q-Learning integrates trust-based evaluation with Q-learning; however, it is still confined by its static trust mechanism and lack of zone-awareness. ZRDM+LFPM, though zone-based, neither employ learning nor provides any security feature, which curbs its robustness against attacks. KB Adaptive Routing lean on rule-based logic for anomaly detection but lacks clustering and adaptability to dynamic environments. ECC Routing assures cryptographic security through elliptic curve encryption, yet it incurs significant resource costs due to heavy computation and lacks learning capabilities. HMM Defence applies statistical modelling for anomaly detection, but it is constrained by slow convergence and poor scalability due to the absence of an energy model. DAPV emphasis on provenance-based anomaly detection but also presents verification overhead, impacting its performance in highly mobile networks. Modified Secured AODV addresses wormhole attacks spontaneously and aims for a swift response, but without learning or predictive adaptability. Similarly, Bee Trust AODV employs a bio-inspired trust model however doesnot pose deep learning and zone awareness, lower efficiency and slow convergence. Collectively, RLSRP stands out as the most balanced and efficient protocol, integrating intelligent learning, zone-awareness, and security in a resource-efficient and scalable manner, making it highly appropriate for practical deployment in dynamic MANET environments. The proposed RLSRP efficiently leverages adaptive clustering to radically curtail routing overhead O(k log n), setting it apart from other protocols that burden themselves with expensive network-wide coordination or flooding. Its strategic use of Deep Q-Networks assures rapid convergence within approximately 1000 episodes, preserving swift route optimisation in dynamic environments. Moreover, RLSRP employs latency deviation for anomaly detection, omitting the need for costly trust or cryptography-based security measures, as a result, substantially reducing overall security costs. This persuasive evaluation demonstrates RLSRP’s superiority in scalability, responsiveness, and efficient security.

### Conclusion

The study introduces the Reinforcement Learning Based Secure Routing Protocol (RLSRP), marking a notable advancement in improving the security and routing efficiency of MANETs by strategically leveraging Deep Q-Networks (DQN) for real-time, intelligent decision-making by dynamically determining optimal routes and effectively countering wormhole attacks. RLSRP remarkably enhance the packet delivery ratio, reduces average end-to-end latency, and ensures better residual energy levels across the network. The protocol exhibits strong scalability, maintaining performance even as the network size increases, especially when compared to existing routing protocols. Moreover, the integration of Adaptive K-hop clustering optimises node selection, stimulating a more resilient and versatile routing environment. Although this approach does entail a slight computational overhead due to the reinforcement learning model’s training and decision-making processes, to avoid unnecessary complexity, we negate cryptography for secure routing. Rather, our DQN-based approach adapts dynamically to emerging threats, negating the need for additional intervention and reliance on encryption. Our proposed method achieves remarkable results, demonstrating superior performance and effectiveness. However, appending additional encryption techniques unnecessarily triggers a trade-off, as it demands more memory and processing power to manage state-action pairs and Q-values, which is still challenging, as excessive computational overhead leads to more complexity because of high processing power and may present challenges in resource-constrained environments, which we plan to address in future work. Excessive clustering may lead to increased routing overhead for incredibly large networks (10+ million nodes). Memory and storage requirements could be a bottleneck. Also, the disparity between exploration and exploitation can sway the convergence time, necessitating further calibration for ideal performance in large-scale and dynamic networks. Our future research will focus on some powerful clustering techniques for better load balancing, thereby achieving more refinement in terms of scalability; also on optimising DQN for low-power devices, incorporating post-quantum cryptography (PQC) to secure control messages, and implementing real-world testbeds to evaluate protocol robustness under practical conditions.

## Limitations and future work

The proposed Reinforcement Learning-Based Secure Routing Protocol (RLSRP) shows big improvements in network lifetime, latency, and data accuracy through a lot of simulation-based analysis. However, there are still some problems. The current validation is confined to a simulation environment, and real-world implementation on extensive MANET testbeds was not achievable within the parameters of this study. Future research will concentrate on the implementation of RLSRP in practical contexts, utilising hardware testbeds and emulation platforms to ascertain its robustness in real-world conditions. In addition, our benchmarking includes a number of current reinforcement learning and clustering-based protocols. However, future studies will compare these protocols to a larger group of current MANET routing protocols. Adding these improvements will make the suggested method more useful and applicable in a wider range of fields, such as military communication, vehicular networks, and disaster recovery systems.

## Data Availability

All data generated or analysed during this study are included in this published article.
